# STY12, a Novel NQO1/HDAC Dual-Targeting Agent, Exhibits Potent Anti-Pancreatic Cancer Activity by ROS-Mediated DNA Damage

**DOI:** 10.3390/biom16060812

**Published:** 2026-05-30

**Authors:** Tong Shen, Xiaojuan Yang, Zhenhua Han, Yanjie Dong, Liqiang Wu

**Affiliations:** 1 School of Pharmacy, Henan Medical University, Xinxiang 453003, China; 2 School of Pharmacy, Xinxiang University, Xinxiang 453003, China; 3 West China School of Medicine, Sichuan University, Chengdu 610041, China

**Keywords:** HDAC, NQO1, ROS, pancreatic cancer, antitumor

## Abstract

Pancreatic cancer (PC) usually results in poor survival with limited treatment options. Reactive oxygen species (ROS) play a key role in the action of HDAC inhibitors. In combination with a ROS generator, it can increase sensitivity to HDAC inhibitors and also overcome resistance to HDAC inhibitors. NQO1-bioactivatable drugs are efficient ROS generators. Therefore, to reduce HDAC inhibitor resistance and enhance its anti-pancreatic cancer activity, in this study, we reported a novel NQO1/HDAC dual-targeting agent, **STY12**, which exhibited potent anti-pancreatic cancer activity through ROS-mediated DNA damage. **STY12** strongly inhibited HDAC1 and HDAC6 activity (IC_50_ = 29 nM and 10 nM, respectively) and exhibited excellent reduction rates by NQO1 (k_cat_/K_m_ = 5.59 × 10^6^ M^−1^s^−1^). **STY12** showed good anti-proliferative effects on PC MIA PaCa-2, SW1990, and Capan-2 cells, with IC_50_ values of 0.23 ± 0.01, 0.25 ± 0.01 and 0.14 ± 0.02 μM, respectively, and lower anti-proliferative effects against normal hTERT-HPNE and BEAS-2B cells. Mechanistic analysis revealed that **STY12** suppressed the proliferation of MIA PaCa-2 cells by increasing the acetylation of histone 3 (H3) and α-tubulin, and increasing ROS-mediated DNA damage. Moreover, **STY12** arrested the cell cycle at the S phase, inhibited the metastasis of MIA PaCa-2 cells, and promoted their apoptosis. Moreover, compared to SAHA and β-Lap, **STY12** exhibited prominent in vivo antitumor activity with negligible toxic effects. Therefore, **STY12** can serve as an effective NQO1/HDAC dual-targeting agent for the treatment of PC.

## 1. Introduction

Pancreatic cancer (PC) is among the most lethal malignancies globally and is characterized by a five-year survival rate of less than 10% and a steadily increasing incidence, thereby constituting a significant global health burden [1,2]. Currently, effective treatment options for PC are quite limited [3,4]. Combination chemotherapy can increase the overall survival rate of patients, but adverse reactions to this treatment can negatively affect the quality of life of patients [5]. Therefore, new and efficient treatment strategies need to be developed to manage PC [6,7].

**Figure 1:**
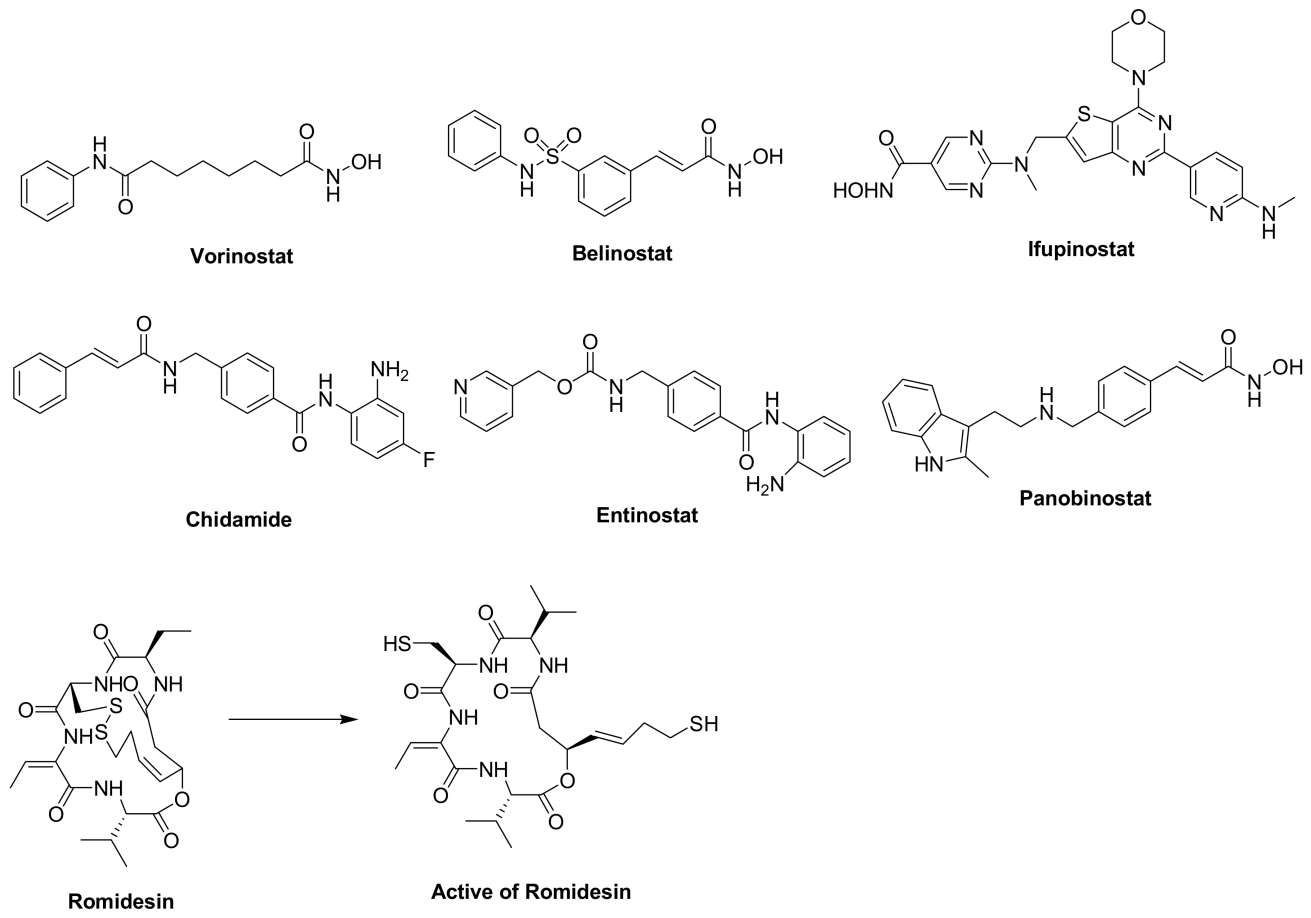
Clinically approved HDACis.

Histone deacetylases (HDACs) are critical epigenetic enzymes. They can remove acetyl groups from histones and lead to chromatin condensation, which is closely associated with cell growth, metastasis, angiogenesis, and gene transcription [8,9]. Researchers have identified 11 zinc-dependent human HDAC isoforms, which are classified as class I (HDAC1, 2, 3, 8), class IIa (HDAC4, 5, 7, 9), class IIb (HDAC6, 10), and class IV (HDAC11) [10,11,12]. HDACs are overexpressed in PC [13] and other human malignancies [14], and they promote the growth and development of tumors. Therefore, HDACs represent optimal antitumor therapeutic targets, and many HDAC inhibitors (HDACis) have been developed [15]. Seven HDACis, including vorinostat, belinostat, romidepsin, panobinostat, chidamide, entinostat, and ifupinostat, have been approved for clinical application (Figure 1) [16,17,18,19,20,21,22]. However, their clinical application as single agents is limited and often associated with various adverse reactions [23,24,25]. Consequently, new HDACis with broad applications and diverse mechanisms need to be developed urgently.

**Figure 2:**
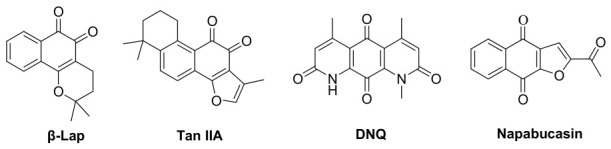
Structures of representative NQO1 substrates.

Tumor cells are characterized by a high content of reactive oxygen species (ROS), increasing the susceptibility to oxidative stress [26]. Pharmacologically increasing the intracellular ROS content via small molecules can serve as an efficient antitumor treatment strategy effective also in certain types of drug-resistant cancer [27,28,29,30]. The cytoplasmic flavoenzyme NAD(P)H quinone oxidoreductase 1 (NQO1) catalyzes the two-electron reduction of quinone compounds to hydroquinones, with the reduced pyridine nucleotide NADPH or NADH serving as the cofactor [31]. Many efficient NQO1 substrates, such as β-lapachone (β-Lap) [32], tanshinone IIA (Tan IIA) [33], deoxynyboquinone (DNQ) [34], and napabucasin [35] (Figure 2), can rapidly generate large amounts of toxic ROS via redox cycling of hydroquinones with the corresponding quinone substrates in NQO1-overexpressing cells. Thus, they may be effective ROS generators for treating cancers [36]. The expression of NQO1 is 5–12 times greater in PC cells than in matched non-carcinoma samples; therefore, NQO1 is a candidate anti-PC therapeutic target [37].

**Figure 3:**
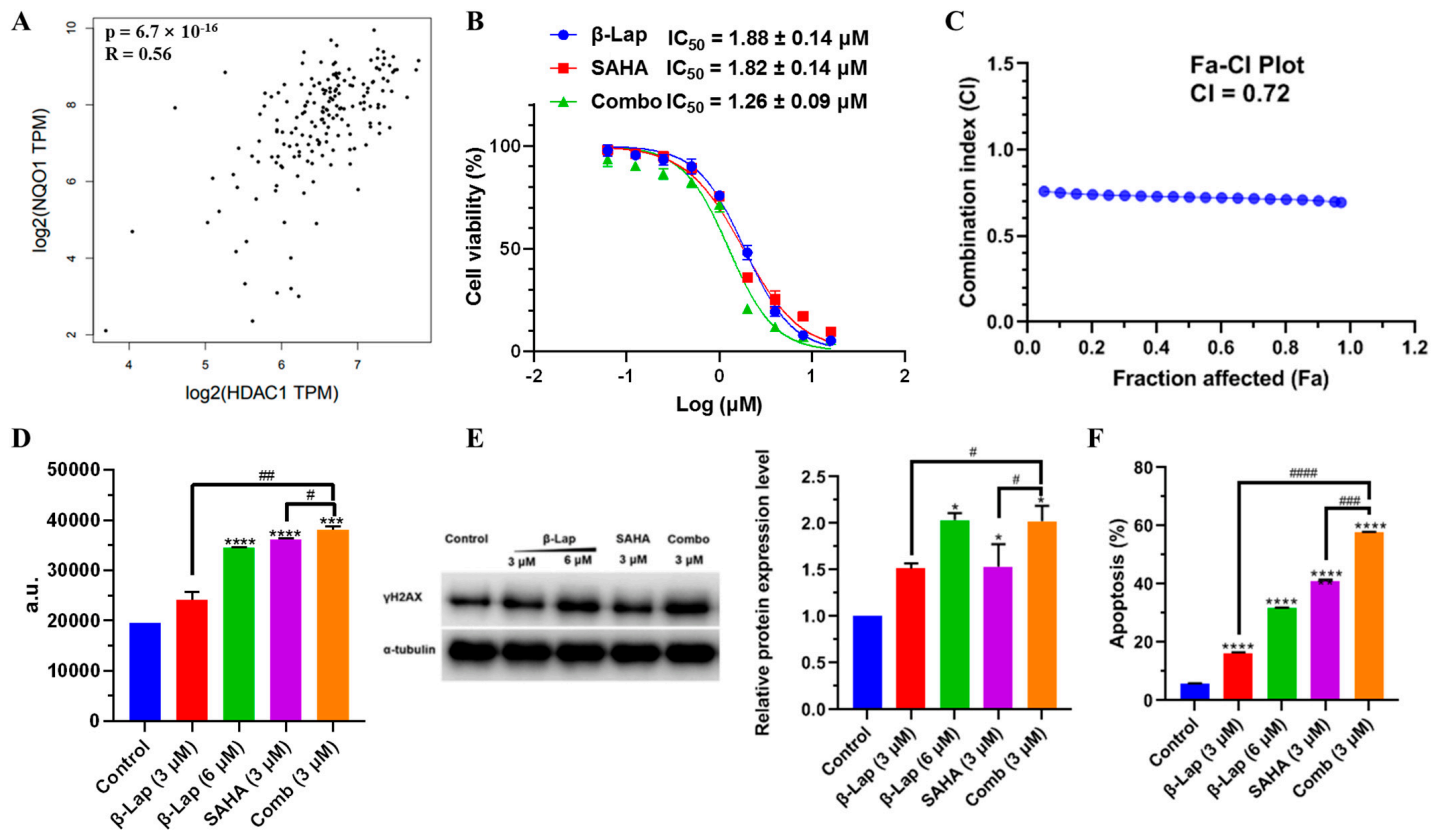
Synergistic effects of β-Lap and SAHA. (**A**) Bioinformatics analysis was carried out using Gene Expression Profiling Interactive Analysis “http://gepia2.cancer-pku.cn/#index (accessed on 21 April 2026)”. Correlation between HDAC1 and NQO1 gene expression in PC patients from The Cancer Genome Atlas (TCGA) database. (**B**) Synergistic effects of β-Lap and SAHA on MIA PaCa-2 cells. The IC_50_ values were calculated after MIA PaCa-2 cells were incubated with the tested compounds (0.625–16 μM) for 72 h. (**C**) Drug combination index of β-Lap and SAHA. (**D**) ROS production capacity after 2 h of treatment with 3 μM and 6 μM β-Lap, 3 μM SAHA, or 3 μM β-Lap + 3 μM SAHA (Combo) in MIA PaCa-2 cells. a.u.: Absorbance Unit. (**E**) Changes in DNA damage marker γH2AX and α-tubulin after 2 h of treatment with 3 μM and 6 μM β-Lap, 3 μM SAHA, or 3 μM β-Lap + 3 μM SAHA (Combo) in MIA PaCa-2 cells. (**F**) Apoptosis induction capacity after 24 h of treatment with 3 μM and 6 μM β-Lap, 3 μM SAHA, or 3 μM β-Lap + 3 μM SAHA (Combo) in MIA PaCa-2 cells. Data were analyzed using GraphPad Prism 8.0, and results are expressed as the means ± SD. * *p* < 0.05, *** *p* < 0.001 and **** *p* < 0.001 vs. the control group. # *p* < 0.05, ## *p* < 0.01, ### *p* < 0.001 and #### *p* < 0.0001 vs. the combo group. Original western blots can be found at Supplementary Materials.

Many researchers have suggested that ROS play important roles in the action of HDACis [38], and that the combination of ROS with a redox-modulating compound can increase their sensitivity to HDACis and overcome resistance to HDACis [39,40,41,42]. Therefore, in the study, we hypothesized that, compared with monotherapeutic regimens, NQO1 bioactivatable drugs combined with HDACis can amplify oxidative stress and have increased antitumor effects. The results of bioinformatic analyses revealed that the expression of NQO1 was strongly correlated with the expression of HDAC1 in PC patients (Figure 3A). In this study, we subsequently analyzed how the HDACi SAHA, the NQO1 substrate β-Lap, and their combination affect the viability of MIA PaCa-2 cells. Compared with the respective monotherapies, SAHA combined with β-Lap had better anti-proliferative effects, with a combination index (CI) of 0.72, which suggested that the synergistic effects were moderate (Figure 3B,C). Additionally, compared with the corresponding monotherapies, SAHA combined with β-Lap slightly increased ROS production (Figure 3D) and potently induced DNA damage-related apoptosis (Figure 3E,F). These findings suggest that targeting HDACs and NQO1 together is advantageous for treating PC. Based on these findings, we proposed the preparation of an NQO1/HDAC dual-targeting agent to reduce HDACi resistance and enhance its anti-pancreatic cancer activity.

## 2. Materials and Methods

### 2.1. Chemistry

#### 2.1.1. General

All reactions were conducted using commercially available reagents without further purification. ^1^H NMR and ^13^C NMR spectra were recorded on a Bruker AV-400 (Bruker, Rheinstetten, Germany) with tetramethylsilane (TMS) used as the internal standard. Chemical shifts were reported in ppm (δ). High–resolution mass spectra were obtained on a Bruker micrOTOF-QIII mass spectrometer (Bruker, Rheinstetten, Germany) using electron spray ionization (ESI) as the ion source. Melting points were determined on an X-4 melting point apparatus (Shanghai Precision Scientific Instruments Co., Ltd, Shanghai, China) without correction.

#### 2.1.2. Synthesis of 3-Methylnaphtho[1,2-*b*]furan-4,5-dione (**2**)

To a solution of 2-hydroxy-1,4-naphthoquinone (174 mg, 1 mmol) in dry chlorobenzene (5 mL), chloroacetone (463 mg, 5 mmol) and NH_4_OAc (77 mg, 1 mmol) were added, and the resulting mixture was heated at 120 °C for 10 h in the dark. After completing the reaction, the mixture was poured into 10 mL of water and extracted with ethyl acetate (3 × 20 mL). The combined organic extracts were washed with brine, dried over Na_2_SO_4_, filtered, and concentrated under reduced pressure. The resulting crude product was purified by silica gel column chromatography to afford compound **2** (38 mg, 18%) as a dark red solid, m.p. 162–164 °C. ^1^H NMR (400 MHz, CDCl_3_) *δ* 8.06–8.04 (m, 2H), 7.68–7.61 (m, 2H), 7.46–7.42 (m, 1H), 7.26 (d, 1H, *J* = 1.2 Hz), 2.28 (d, 1H, *J* = 1.2 Hz). HRMS-ESI (*m*/*z*): calcd for C_13_H_8_NaO_3_ [M+Na]^+^ 235.0366, found: 235.0365.

#### 2.1.3. Synthesis of 4,5-Dihydro-3-methyl-4,5-dioxonaphtho[1,2-*b*]furan-2-carbaldehyde (**3**)

DMF (0.37 mL, 4.72 mmol) was added dropwise to POCl_3_ (0.43 mL, 4.72 mmol) at 0 °C. Then, compound **2** (100 mg, 0.47 mmol) in DMF was added, and the resulting mixture was heated at 65 °C for 4 h. After completing the reaction, the mixture was poured into ice water, and the saturated Na_2_CO_3_ solution was used to adjust the pH to 9. The pH is finally adjusted to neutral with the addition of HCl. The precipitate was filtered and was purified by silica gel column chromatography to afford compound **3** (36 mg, 15%) as an orange solid, m.p. 196–198 °C. ^1^H NMR (400 MHz, CDCl_3_) *δ* 9.91 (s, 1H), 8.16 (dd, 1H, *J* = 7.6, 1.2 Hz), 7.95 (dd, 1H, *J* = 8.0, 1.2 Hz), 7.81–7.14 (m, 1H), 7.64–7.60 (m, 1H), 2.69 (s, 3H). HRMS-ESI (*m*/*z*): calcd for C_14_H_8_NaO_4_ [M+Na]^+^ 263.0315, found: 263.0303.

#### 2.1.4. Synthesis of 4,5-Dihydro-3-methyl-4,5-dioxonaphtho[1,2-*b*]furan-2-carboxylic Acid (**4**)

A mixture of compound **3** (40.8 mg, 0.17 mmol), 30% H_2_O_2_ (0.07 mL, 0.68 mmol), NaH_2_PO_4_ (82 mg, 0.68 mmol) in 2 mL H_2_O, and NaClO_2_ (15 mg, 0.17 mmol) in CH_3_CN (10 mL) was stirred at room temperature for 8 h. Then the solvent was partly distilled, and the precipitate was filtered. The obtained compound was used in the next step without further purification.

#### 2.1.5. Syntheis of (9*H*-Fluoren-9-yl)methyl 6-(Tetrahydro-2*H*-pyran-2-yloxycarbamoyl) Hexylcarbamate (**6**)

To a stirred solution of Fmoc-7-Ahp-OH compound **5** (367 mg, 1 mmol,) and *O*-(tetrahydro-2*H*-pyran-2-yl)hydroxylamine (328 mg, 2.8 mmol) in 20 mL of dry CH_2_Cl_2_, DIPEA (3 mmol, 387 mg), EDCI (1.3 mmol, 248 mg) and HOBt (1.3 mmol, 176 mg) were added at 0 °C. Then the reaction mixture was stirred for 12 h at room temperature. After completing the reaction, the mixture was poured into 20 mL water and extracted with CH_2_Cl_2_ (3 × 20 mL). The combined organic layer was washed with brine, dried over anhydrous Na_2_SO_4_, filtered, and then evaporated under reduced pressure. The resulting crude product was purified by silica gel column chromatography to afford compound **6** (345 mg, 77%) as a white solid, m.p. 103–105 °C. ^1^H NMR (400 MHz, DMSO-*d*_6_) *δ* 10.92 (s, 1H), 7.89 (d, 2H, *J* = 7.6 Hz), 7.69 (d, 2H, *J* = 7.6 Hz), 7.43–7.26 (m, 5H), 4.80 (S, 1H), 4.41–4.19 (m, 3H), 3.94–3.89 (m, 1H), 3.51–3.47 (m, 1H), 2.98–2.93 (m, 2H), 1.97 (t, 2H, *J* = 7.2 Hz), 1.67–1.21 (m, 14H). HRMS-ESI (*m*/*z*): calcd for C_27_H_34_N_2_NaO_5_ [M+Na]^+^ 489.2360, found: 489.2362.

#### 2.1.6. Synthesis of 7-Amino-*N*-(tetrahydro-2*H*-pyran-2-yloxy)heptanamide (**7**)

To a stirred solution of compound **6** (1 mmol, 466 mg) in 20 mL of CH_2_Cl_2_ at room temperature, 10 mL of diethylamine was added, and the reaction mixture was stirred for 2 h. After completing the reaction, the volatiles were evaporated under reduced pressure to yield the crude product, which was purified by flash column chromatography to afford the desired amine compound **7** (13 mg, 5.3%) as a clear liquid. ^1^H NMR (400 MHz, DMSO-*d*_6_) *δ* 7.75–7.73 (m, 1H), 4.89–4.71 (m, 1H), 3.94–3.89 (m, 1H), 3.50–3.47 (m, 1H), 3.02–2.97 (m, 1H), 2.04–1.94 (m, 3H), 1.67–1.23 (m, 16H). HRMS-ESI (*m*/*z*): calcd for C_12_H_25_N_2_O_3_ [M+H]^+^ 245.1860, found: 245.1854.

#### 2.1.7. Synthesis of *N*-(6-(Tetrahydro-2*H*-pyran-2-yloxycarbamoyl)hexyl)-4,5-dihydro-3-methyl-4,5-dioxonaphtho[1,2-*b*]furan-2-carboxamide (**8**)

To a stirred solution of compound **4** (1 mmol, 256 mg) in CH_2_Cl_2_ (10 mL), compound **7** (1 mmol, 245 mg), DIPEA (3 mmol, 387 mg), EDCl (1.3 mmol, 248 mg), and HOBt (1.3 mmol, 176 mg) were added, and the reaction mixture was stirred for 12 h at room temperature. After completing the reaction, the mixture was poured into 20 mL water and extracted with CH_2_Cl_2_ (3 × 20 mL). The combined organic layers were washed with saturated NaHCO_3_ and brine, dried over anhydrous Na_2_SO_4_, filtered, and concentrated under reduced pressure. The crude product was purified by silica column chromatography to give compound **8** (68 mg, 14%) as an orange-red solid, m.p. 98–100 °C. ^1^H NMR (400 MHz, DMSO-d6) *δ* 10.90 (s, 1H), 8.61 (t, 1H, *J* = 6.0 Hz), 8.06 (dd, 1H, *J* = 7.6, 1.2 Hz), 7.96 (dd, 1H, *J* = 7.6, 1.2 Hz), 7.84–7.80 (m, 1H), 7.63–7.58 (m, 1H), 4.80 (s, 1H), 3.94–3.88 (m, 1H), 3.51–3.46 (m, 1H), 3.28–3.23 (m, 2H), 2.50–2.49 (m, 2H), 2.01–1.95 (m, 2H), 1.67–1.23 (m, 15H). ^13^C NMR (100 MHz, DMSO-d6) *δ* 179.60, 175.64, 169.59, 158.58, 143.68, 135.23, 131.21, 130.46, 129.64, 127.68, 125.99, 123.38, 122.16, 101.27, 93.97, 63.18, 38.81, 32.76, 29.62, 28.24, 26.67, 25.60, 25.15, 20.88, 18.75, 10.09. HRMS-ESI (*m*/*z*): calcd for C_26_H_30_N_2_NaO_7_ [M+Na]^+^ 505.1945, found: 505.1960.

#### 2.1.8. Synthesis of *N*-(6-(Tetrahydro-2*H*-pyran-2-yloxycarbamoyl)hexyl)-4,5-dihydro-3-methyl-4,5-dioxonaphtho[1,2-*b*]furan-2-carboxamide (**STY12**)

A mixture of compound **8** and 4 M HCl in 1,4-dioxane (10 mL) was stirred for 8 h at room temperature. After completing the reaction, the volatiles were evaporated under reduced pressure to yield the crude product, which was washed with CH_2_Cl_2_ (3 × 20 mL) to give **STY12** (191 mg, 48%) as a red solid, m.p. 132–134 °C. ^1^H NMR (400 MHz, DMSO-*d*_6_) *δ* 10.39 (s, 1H), 8.71–8.65 (m, 2H), 8.07 (d, 1H, *J* = 7.6 Hz), 7.96 (d, 1H, *J* = 7.6 Hz), 7.82 (t, 1H, *J* = 7.2 Hz), 7.61 (t, 1H, *J* = 7.6 Hz), 3.26 (d, 2H, *J* = 6.8 Hz), 1.96 (t, 2H, *J* = 7.2 Hz) 1.57–1.23 (m, 11H). ^13^C NMR (101 MHz, DMSO-*d*_6_) *δ* 179.58, 175.60, 169.59, 158.54, 158.46, 143.68, 135.22, 131.18, 130.43, 129.61, 127.67, 125.93, 123.42, 122.14, 38.81, 32.69, 29.65, 28.83, 26.71, 25.56, 10.09. HRMS-ESI (*m*/*z*): calcd for C_21_H_22_N_2_NaO_6_ [M+Na]^+^ 421.1370, found: 421.1362. Purity: 99.54% (HPLC, *t*_R_ = 8.024 min).

### 2.2. Cell Culture

MIA PaCa-2 (Cat#: KGG3357-1), SW199 (Cat#: KGG3223-1), Capan-2 (Cat#: KGG3359-1), and HTERT-HPNE (Cat#: KGG3111-1) cells were obtained from Jiangsu KeyGEN BioTECH Corp., Ltd. (Nanjing, China). BEAS-2B cells (Cat#: STCC10202P-1) were acquired from the Wuhan Zishan Biotech Corp., Ltd. (Wuhan, China). MIA PaCa-2, HTERT-HPNE, and BEAS-2B cells were cultured in DMEM medium supplemented with 10% fetal bovine serum (FBS) and 1% penicillin–streptomycin, while SW199 and Capan-2 were cultured in RPMI-1640 medium supplemented with 10% FBS and 1% penicillin–streptomycin. Take cells with good growth status to be tested, and when they reach convergence, add 1 mL of 0.25% trypsin solution to the culture bottle. Digest in a 37 °C incubator until the cells are close to detachment from the bottle wall. Suck out the digestion solution, add new culture medium, gently blow to prepare a cell suspension, and count. All the cells were kept in a humidified incubator with 5% CO_2_ at 37 °C.

### 2.3. Cell Viability

Initially, MIA PaCa-2, SW1990, Capan-2, BEAS-2B and HTERT-HPNE cells at a density of 3.5 × 10^4^, 5 × 10^4^, 3.5 × 10^4^, 3.5 × 10^4^ and 3.5 × 10^4^ cells/mL, respectively, were seeded in 96-well plates overnight for culture. Then, they underwent compound treatment at the concentration gradient (0.01–33 µM, using DMSO as a control) for 72 h. Subsequently, cells were treated with MTT solution (50 μL/well) for 4 h at 37 °C. Cell viability was analyzed by measuring OD using a multimode microplate reader at 490 nm. Cell viability was calculated as the following equation: [OD(compound) − OD(blank)]/[OD(control) − OD(blank)] × 100%.

### 2.4. In Vitro HDAC Inhibition Assay

Enzymatic inhibitory activities were identified for HDAC1, HDAC4, HDAC6, HDAC8, HDAC11 (Signalchem, Richmond, BC, Canada), Ac-Leu-GlyLys(Ac)-AMC, and Ac-Leu-Gly-Lys (Tfa)-AMC (Acmec, Shanghai, China). Every enzymatic reaction lasted for 30 min at 37 °C and used DMSO as a control. The reaction system (50 µL) consisted of 25 mM Tris (pH 8.0), 137 mM NaCl, 2.7 mM KCl, 1 mM MgCl_2_, 0.1 mg/mL BSA, and HDAC, along with the enzyme substrate. After dilution with 10% DMSO, the compound (5 µL) was introduced into the reaction mixture (50 µL) to ensure that the final DMSO concentration was 1% and the compound concentration was 0.1 nM–10 µM during every reaction. To conduct this assay, the fluorescent product level was quantified in the solution after the enzymatic reaction. Next, fluorescence was examined using a SpectraMax M5 microtiter plate reader (Molecular Devices, San Jose, CA, USA) at excitation and emission wavelengths of 350–360 nm and 450–460 nm, respectively.

### 2.5. In Vitro NQO1 Assay

Compounds were monitored as NQO1 substrates using an NADPH recycling assay and recombinant NQO1 (Cat#: D1315, Sigma, Darmstadt, Germany), in which NADPH oxidation to NADP^+^ was monitored by absorbance (A340 nm) on a UV-Vis spectrophotometer (Edinburgh Instruments, Livingston, UK, using DMSO as a control). After the compounds were dissolved in DMSO (final concentration was 1.25–40 µM during every reaction), 400 μM NADPH or 1.4 μg/mL NQO1 in phosphate-buffered saline (PBS) buffer (10 mM, pH 7.4) was added to each well. To initiate the enzymatic reaction, NADPH solution was automatically dispensed into each well, and the changes in absorbance were recorded every 5 s within a period of 5 min at ambient temperature. The linear curve of absorbance vs. time (initial 20 s to 1 min) was plotted; next, the velocity was determined.

### 2.6. Molecular Docking

Molecular docking studies were conducted using the AutoDock Vina 1.2.3 software. The HDAC1 (PDB: 4BKX), HDAC6 (PDB: 5EEI), and NQO1 (PDB ID: 2F1O) crystal structures were obtained from the RCSB Protein Data Bank. The prepared **STY12** was docked into the binding sites of HDAC1 (PDB: 4BKX), HDAC6 (PDB: 5EEI), and NQO1 (PDB ID: 2F1O), and the binding modes were analyzed using PyMOL2.5.

### 2.7. Western Blotting

Initially, MIA PaCa-2 cells (1 × 10^7^ cells/mL)were seeded in 12-well plates and treated with **STY12** (0.2, 1 μM), **STY12** (1 μM) + NAC (3 mM), SAHA (1 μM) or β-Lap (1 μM) for 48 h. Then, the cells were collected and rinsed with PBS. Next, the lysis solution was added for 15 min, and the sample was centrifuged to obtain the protein mixture. Proteins were separated through 10–15% SDS-PAGE, followed by transfer to polyvinylidene difluoride (PVDF) membranes. After the membranes were blocked for 1 h with 5% non-fat milk, they were incubated with primary antibodies overnight. After being washed with PBS buffer for 1 h, the membranes were probed with secondary antibodies. Finally, an ECL reagent was used to visualize the signal. Antibodies, including Acetyl-Histone H3 (Lys9) Rabbit pAb (Cat#: 340016), Acetyl-α-Tubulin (Lys40) Monse mAb (Cat#: 250236), and Phospho-Histone H2AX (Ser139) Rabbit mAb (Cat#: R381558) and α-Tubulin Rabbit mAb (Cat#: R23452), were obtained from Chengdu Zen Bioscience Co., Ltd. (Chengdu, China). Rabbit Anti-GAPDH pAb (Cat#: 3KGC6102-1) was obtained from Jiangsu KeyGEN BioTECH Corp., Ltd. (Nanjing, China).

### 2.8. Measurement of Intracellular ROS Levels

Measurements of intracellular ROS levels were determined using a reactive oxygen species assay kit (Cat#: KGA7308-100, KeyGEN BioTECH, China). Briefly, MIA PaCa-2 cells (3 × 10^5^ cells/mL) were seeded in 6-well plates and incubated with **STY12** (0.2, 1 μM), **STY12** (1 μM) + DIC (50 μM), SAHA (1 μM) or β-Lap (1 μM) for 48 h. After drug treatment, the cells were incubated with 10 μM DCFH-DA for 30 min at 37 °C, washed with PBS twice, and immediately visualized via laser scanning microscopy and analyzed via flow cytometry (FACScan flow cytometer, Becton Dickinson, Franklin Lakes, NJ, USA).

### 2.9. Apoptosis Assay

Apoptosis was determined using the Annexin V-FITC/7-AAD Apoptosis Kit (Cat#: KGA1106-50, KeyGEN BioTECH, Nanjing, China). Initially, MIA PaCa-2 cells (3 × 10^5^ cells/mL) were seeded in 6-well plates and incubated with **STY12** (0.2, 1 μM), SAHA (1 μM), β-Lap (1 μM) or SAHA (1 μM)+ β-Lap (1 μM) for 48 h. Then, the cells were collected and rinsed with PBS. Next, 500 μL of binding solution, 5 μL of Annexin V-APC staining solution, and 5 μL of 7-AAD staining solution were added to the samples, which were incubated for 15 min in the dark. Finally, the samples were analyzed via flow cytometry.

### 2.10. Cell Cycle Assay

Cell cycle assay was performed by PI/RNase reagent (Cat#: KGA9101-50, KeyGEN BioTECH, Nanjing, China). First, MIA PaCa-2 cells (3 × 10^5^ cells/mL) were seeded in 6-well plates and incubated with **STY12** (0.2, 1 μM), SAHA (1 μM), β-Lap (1 μM) or SAHA (1 μM)+ β-Lap (1 μM) for 48 h. Then, the cells were washed once with PBS and incubated overnight in prechilled 70% ethanol at 4 °C. Later, the cells were rinsed with PBS to eliminate ethanol, followed by 30 min of incubation with 50 μL of RNase A and 450 μL of PI at 4 °C. Finally, the samples were analyzed via flow cytometry.

### 2.11. Transwell Assay

The invasive ability of cells was assessed using Transwell chambers (Cat#: 3422, Corning Incorporated, Corning, NY, USA), pre-coated with Matrigel. First, MIA PaCa-2 cells (1 × 10^5^ cells/mL) were seeded in 24-well plates and incubated with **STY12** (0.2, 1 μM), SAHA (1 μM) or β-Lap (1 μM) for 48 h. Then, the cells were washed once with PBS and inoculated in the upper Transwell chamber. The bottom chamber was filled with a medium containing 20% FBS. After being incubated for 48 h at 37 °C, the cells were stained with crystal violet. Three randomly captured fields were photographed, and the cell numbers were quantified using the ImageJ1.35a. 

### 2.12. In Vivo Anti-Cancer Activity

Animals were treated according to the protocols established by the ethics committee of Xinxiang University. BalB/c nude mice (female, 5–6 weeks) were purchased from Sibeifu (Suzhou) Biotechnology Co., Ltd. (Suzhou, China), with an animal license number SYXK(Su)2023-0001. The mice were anesthetized with 3% isoflurane using a rodent anesthesia system (PerkinElmer Instruments Co., Ltd., Shanghai, China). Afterward, the mice were s.c. injected with 5 × 10^7^ MIA PaCa-2 cells in the right flank. When the mean tumor volume reached about 150 mm^3^, the animals were randomly classified into four groups (*n* = 4 in each group) and intraperitoneally administered control solvent, 20 mg/kg napabucasin, 20 mg/kg β-Lap, or 20 mg/kg **STY12** every other day for 18 days. The body weights and tumor sizes were recorded every three days. After 18 days, the mice were dissected, and the tissues were weighed.

### 2.13. In Silico Physicochemical, Pharmacokinetic, and Toxicological Assessments

Physicochemical properties and pharmacokinetic parameters of **STY12** were predicted using the SwissADME web server “https://www.swissadme.ch/ (accessed on 21 April 2026)”.

### 2.14. Stability Study

**STY12** (20 μM) was co-incubated with cell culture media (DMEM or RPMI-1640) at 37 °C for different times. Following centrifugation, the supernatant was analyzed by HPLC to determine the percentage of remaining **STY12**.

### 2.15. Statistical Analysis

Results are shown as means ± standard deviations for the indicated number of independently performed experiments. The statistical significance of the differences in mean values was determined by Student’s *t*-test or ANOVA. * *p* < 0.05, ** *p* < 0.01, *** *p* < 0.001 and **** *p* < 0.0001 represented statistical significance.

## 3. Results

### 3.1. Rational Design of **STY12**

**Figure 4:**
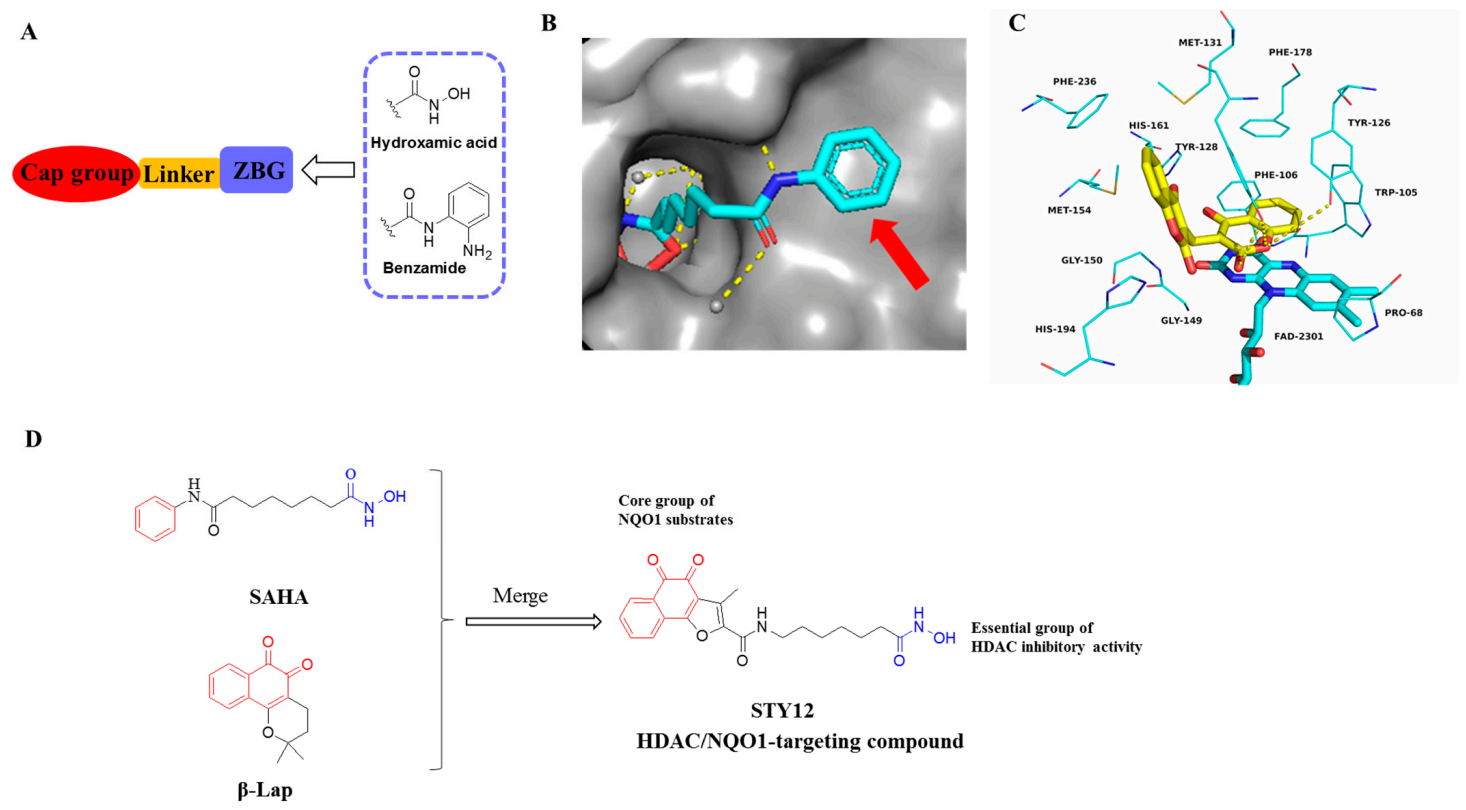
Design strategy for the compound **STY12**. (**A**) The pharmacological structure of HDACi. (**B**) Cocrystal structure of HDAC2-SAHA (PDB ID: 4LXZ). The solvent-exposed regions are highlighted with red arrows. (**C**) DIC (yellow) in the crystal structure (2F1O). (**D**) Design strategy for the compound **STY12**.

Generally, HDACis involve three common pharmacophores: (1) one zinc-binding group (ZBG) chelated with a zinc ion at the bottom portion of the active site of the enzyme; (2) one hydrophobic linker that binds within a narrow tunnel; and (3) one enzyme surface recognition region (Cap) that interacts with the amino acids around the rim of the active site (Figure 4A). Hydroxamic acid and anilinobenzamide are commonly used in ZBG because of their strong chelating effect, whereas the cap region can accommodate structurally diverse groups because of its exposure to the solvent for modulating selectivity among distinct isoforms, enhancing anti-cancer activity or producing multi-target molecules (Figure 4B). By analyzing the crystal structure of NQO1 with dicoumarol (DIC) (PDB ID code: 2F1O), it is found that the binding site for NQO1 substrates is an L-shaped pocket. Notably, the bound ligand DIC also has an L-type molecular shape that fits well with the L-shaped binding pocket of NQO1. β-Lap, an efficient NQO1 substrate, exerts strong antitumor effects via NQO1-induced ROS generation. The 1,2-naphthoquinone moiety in β-Lap is essential for its cytotoxicity. However, β-Lap only occupied the bottom of the pocket parallel to FAD, but missed the side binding pocket. Therefore, using the pharmacophore fusion strategy, we introduced 1,2-naphthoquinone from β-Lap in SAHA as a cap group and designed a novel L-shaped NQO1/HDAC dual-targeting agent, **STY12** (Figure 4C), which increased ROS generation and showed potent anti-PC effects.

### 3.2. Chemistry

**Scheme 1:**
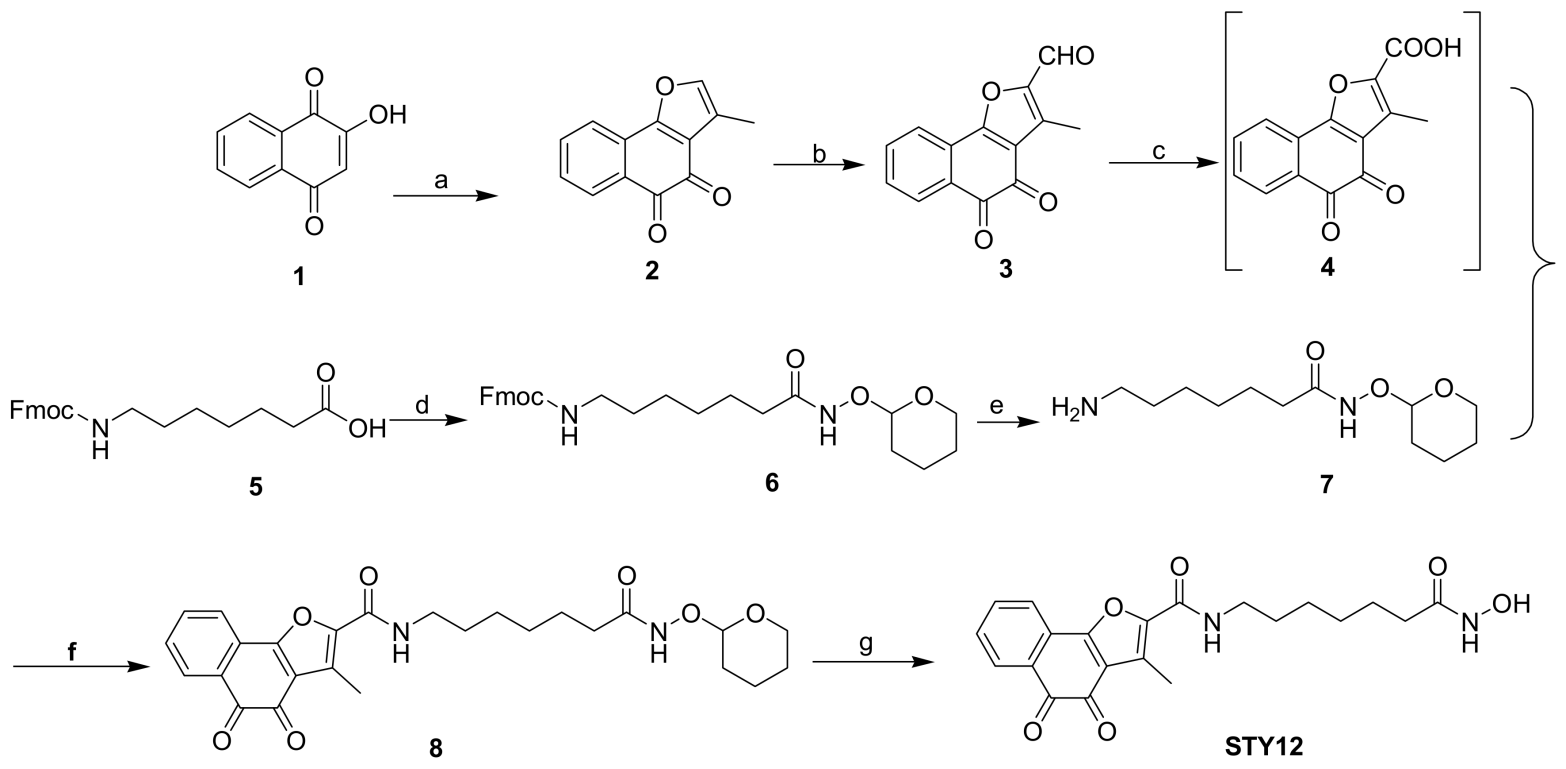
Synthesis of **STY12**. Reagents and conditions: (a) Chloroacetone, NH_4_OAc, PhCl, 120 °C, 18%; (b) DMF, POCl_3_, 65 °C, 15%; (c) H_2_O_2_, NaClO_2_; (d) THP-*O*-NH_2_, EDCl, HOBt, DIPEA, 77%; (e) 30% diethylamine in CH_2_Cl_2_, 5.3%; (f) EDCl, HOBt, DIPEA; (g) 4 M HCl in 1,4-dioxone, 48%.

The **STY12** preparation process is shown in Scheme 1. First, compound **2** was prepared via a Feist-Bénary reaction after treatment with 2-hydroxy-1,4-naphthoquinone and chloroacetone in the presence of ammonium acetate. The Vilsmeier reaction between compound **2** and DMF, as well as POCl_3_, subsequently yielded compound **3**. When sodium chloride and hydrogen peroxide were present, compound **3** was subsequently oxidized to produce compound **4** (carboxylic acid). Second, commercially available fmoc-7-aminoheptanoic acid and *O*-(tetrahydro-2*H*-pyran-2-yl)hydroxylamine (THP-*O*-NH_2_) were used to produce compound **6** with EDCl. Next, the Fmoc protecting group was removed using a 30% solution of diethylamine in CH_2_Cl_2_ to produce compound **7**. Finally, compounds **4** and **7** were subjected to a coupling reaction with EDCI/HOBt in CH_2_Cl_2_ to obtain intermediate **8**, which was subsequently deprotected with 4 M HCl in dioxane to produce **STY12**.

### 3.3. In Vitro Anti-Proliferation Assay

**Figure 5:**
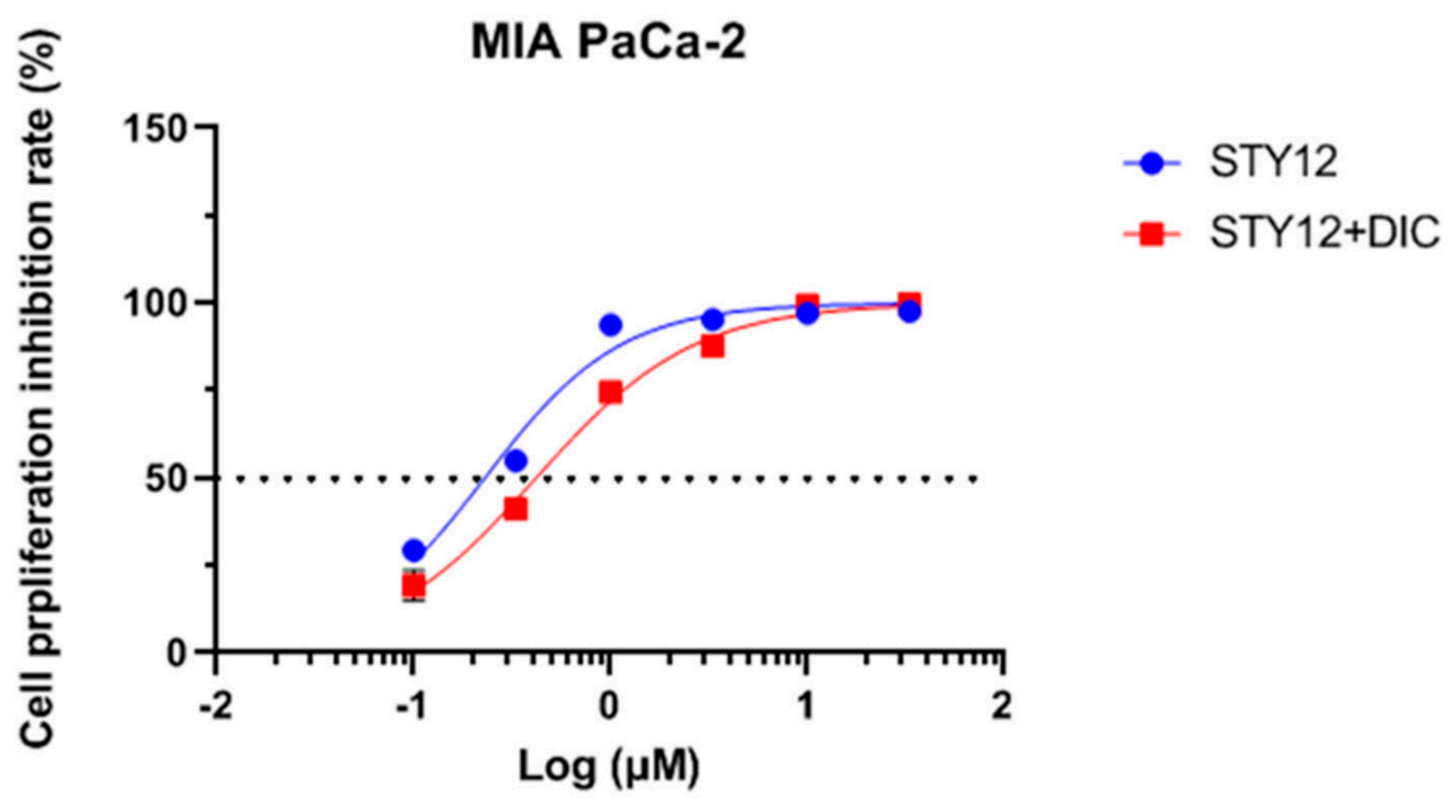
In vitro anti-proliferative activities of compounds **STY12** and **STY12**+DIC against MIA PaCa-2 cells. MIA PaCa-2 cells were treated with **STY12** (0.01–33 μM) and **STY12** (0.01–33 μM) + DIC (50 μM) for 72 h. Data were analyzed using GraphPad Prism 8.0, and results are expressed as the means ± SD of at least two separate determinations.

The NQO1/HDAC dual-targeting agent **STY12** is a new compound whose ability to resist the proliferation of PC cells (MIA PaCa-2, SW1990, and Capan-2) was first analyzed by the MTT assay. SAHA (HDACi) and β-Lap (NQO1 bioactivatable drug), two potent single-target compounds, together with their combination, were used as control drugs. As shown in Table 1, compared with SAHA, β-Lap, and their combination, **STY12** strongly inhibited MIA PaCa-2, SW1990, and Capan-2 cells, with IC_50_ values of 0.23 ± 0.01, 0.25 ± 0.01 and 0.14 ± 0.02 μM, respectively. Given that **STY12** has strong anti-proliferative effects on PC cells, we subsequently evaluated the cytotoxicity of the compound to normal hTERT-HPNE and BEAS-2B cells. Unfortunately, **STY12** exhibited submicromolar activity against the two normal cell lines, indicating a lower safety profile. Moreover, after the NQO1 inhibitor DIC was added, **STY12** weakened the cytotoxic effects on MIA PaCa-2 cells (IC_50_ = 0.49 ± 0.02) (Figure 5), which indicated that NQO1 partially mediated the anti-PC effects of **STY12**.

### 3.4. Compound **STY12** Inhibited HDAC Activity

**Figure 6:**
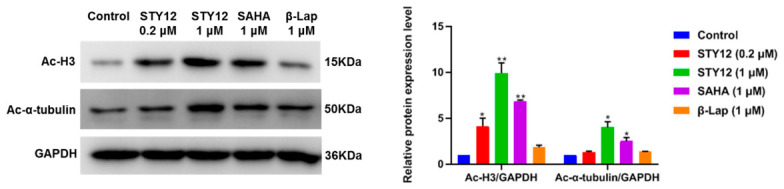
Effects of compounds **STY12** (0.2, 1 μM), SAHA (1 μM) and β-Lap (1 μM) on the levels of Ac-H3 and Ac- a-tubulin in MIA PaCa-2 cells. Cells were treated for 48 h. The results are expressed as the means ± SD. * *p* < 0.05, ** *p* < 0.01 vs. the control group. Original western blots can be found at Supplementary Materials.

After evaluating the ability of **STY12** to prevent the proliferation of PC cells, we first evaluated its ability to inhibit diverse HDAC classes, such as HDAC1, HDAC4, HDAC6, HDAC8, and HDAC11. Compared with SAHA, **STY12** had similar inhibitory activity on HDACs (1 and 6), with IC_50_ values of 29 and 10 nM, respectively (Table 2). Additionally, compared with HDAC4, HDAC7, HDAC8, and HDAC11, **STY12** had more potent inhibitory effects on HDAC1 and HDAC6. Therefore, it is a pan-HDAC inhibitor. We also conducted Western blotting to analyze the functions of **STY12** in histone 3 (H3, HDAC 1/2/3/8 substrate) and the acetylation of α-tubulin (HDAC 6 substrate) in MIA PaCa-2 cells. As shown in Figure 6, compared with SAHA at an identical dose, **STY12** enhanced H3 and α-tubulin acetylation in a dose-dependent manner, with a considerably greater effect on acetylation-induced capacity. Therefore, **STY12** suppresses cellular class I HDACs and HDAC6, confirming the results of the enzyme analysis.

### 3.5. Compound **STY12** Exhibited Excellent Reduction Rates by NQO1

**Figure 7:**
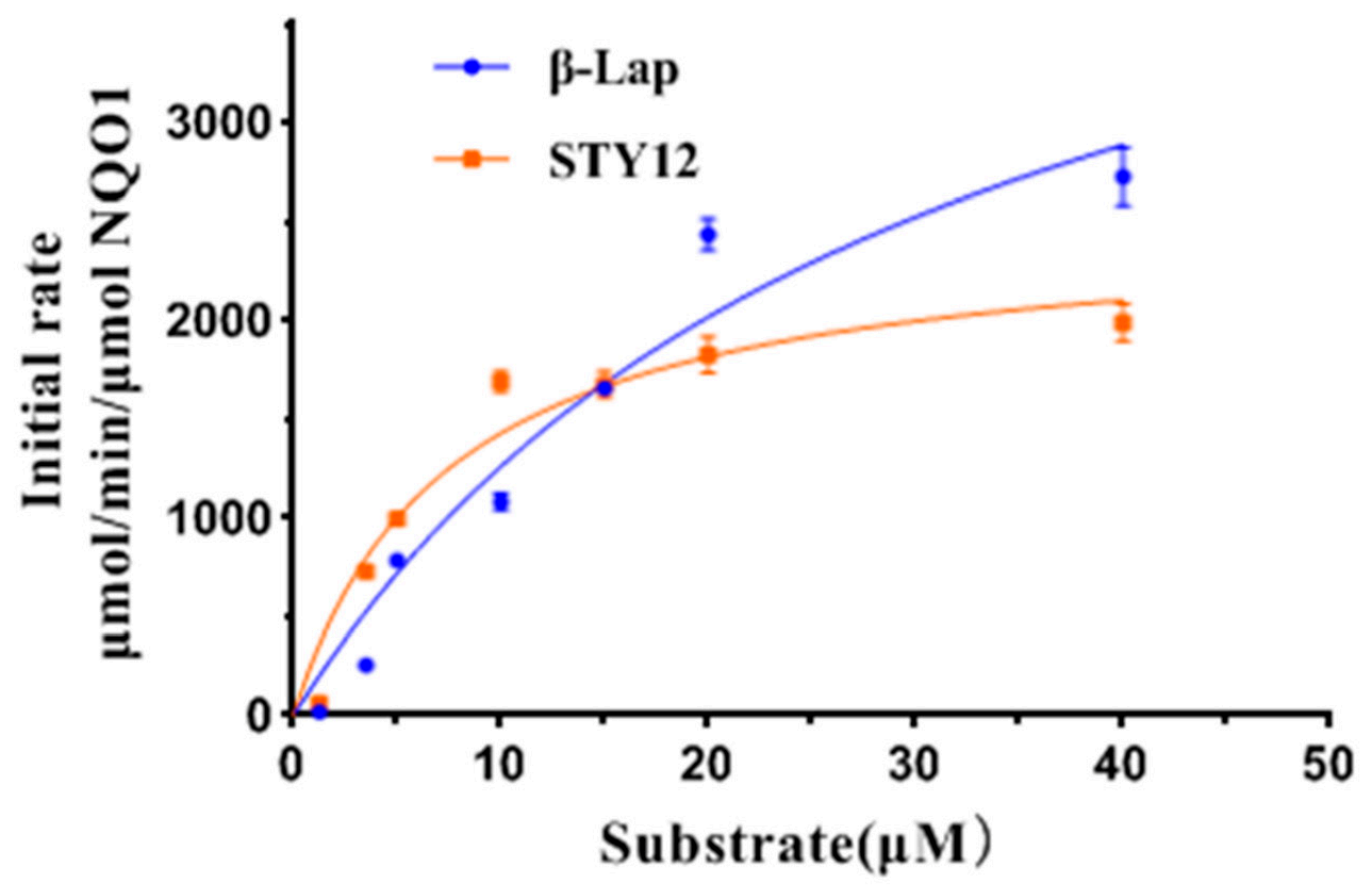
Michaelis–Menten curves for **STY12** and β-Lap with NQO1.

As a cytosolic flavoenzyme, NQO1 is considerably upregulated in PC cells. Quinones have been evaluated as candidate NQO1 substrates that are associated with redox cycling for generating ROS. To assess its role as an NQO1 substrate, the enzymatic conversion rates of NQO1 at different concentrations (0–40 μM) were analyzed. Next, the relevant Michaelis–Menten curves were plotted for **STY12**. As shown in Figure 7, compared with SAHA, **STY12** exhibited enhanced catalytic performance (k_cat_/K_m_ = 5.59 × 10^6^ M^−1^s^−1^ and 2.79 × 10^6^ M^−1^s^−1^, respectively), which indicated that **STY12** has excellent reduction rates via NQO1 and is a potent substrate of NQO1.

### 3.6. Molecular Docking

**Figure 8:**
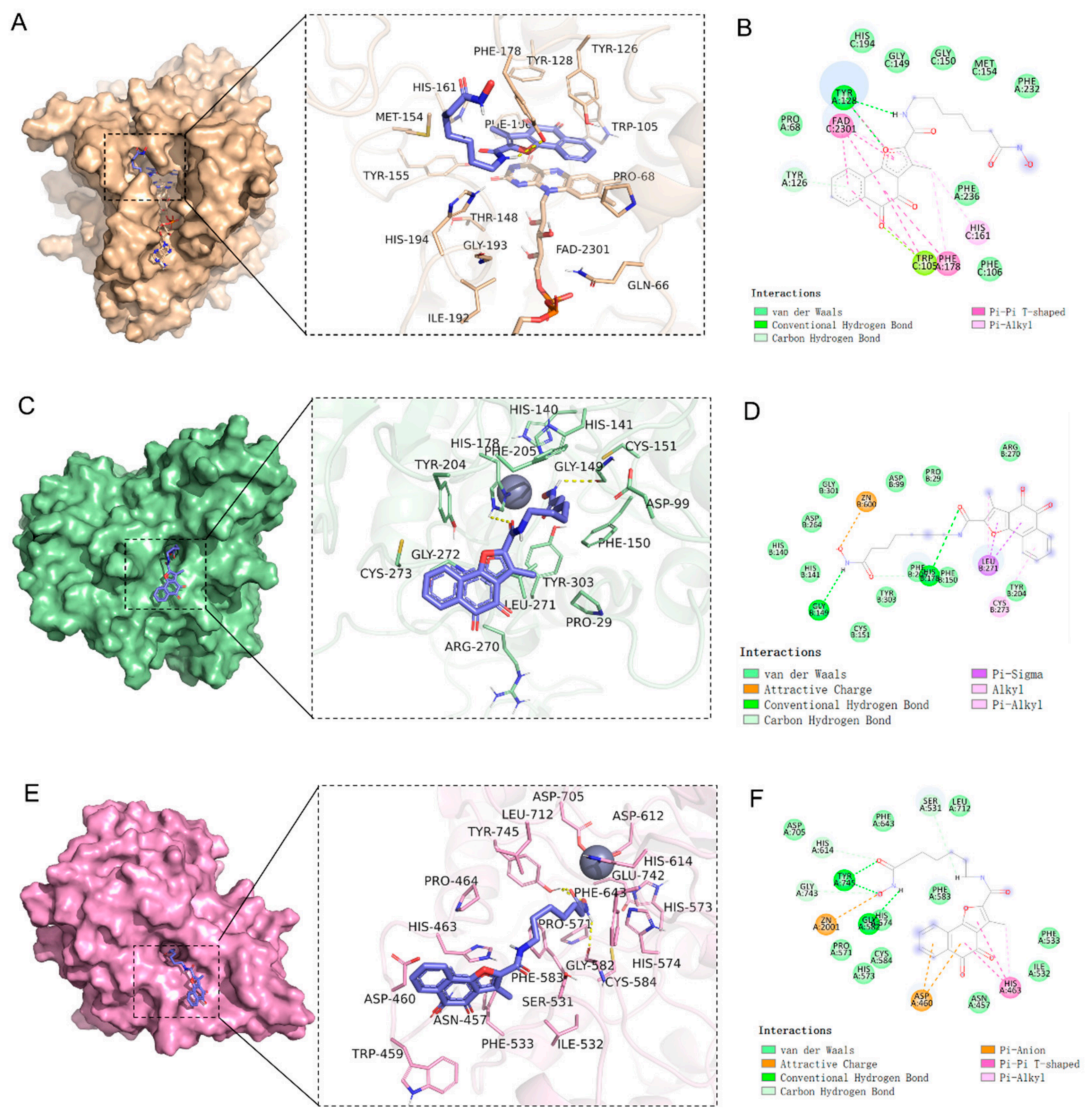
Binding mode of **STY12** with NQO1 (**A**,**B**), HDAC1 (**C**,**D**) and HDAC6 (**E**,**F**), respectively. (**A**,**C**,**E**) The left panel shows the overall view, and the right panel presents the local binding pocket. (**B**,**D**,**F**) Schematic 2D interaction.

Molecular docking enables the convenient and efficient investigation of interactions between target proteins and small molecules. In this study, AutoDock Vina 1.2.3 software was used to conduct docking studies on **STY12** with the proteins NQO1, HDAC1, and HDAC6. As shown in Figure 8A,B, **STY12** formed π-stacking interactions with the FAD isoalloxazine ring and a hydrogen bond with the Tyr128 residue. The C-2 substituent was found to fit well into the side hydrophobic pocket formed by Try128, Met154, His161, and Tyr155. Additionally, the docking score was −9.467 kcal/mol, which indicated that the binding potential of the small molecule with the protein was favorable.

As shown in Figure 8C,D, **STY12** formed hydrogen bonds with Gly149 and His-178 of the HDAC1 protein, confirming its tight binding. Additionally, Zn^2+^ of the HDAC1 protein was chelated by the ZBG of **STY12**. Moreover, small molecules also form hydrophobic interactions with several residues, such as Cys273 and Leu271, providing strong van der Waals forces that further stabilize the complex. The docking score was −6.933 kcal/mol, which indicated that the small molecule had a favorable binding potential with HDAC1.

As shown in Figure 8E,F, **STY12** formed hydrogen bonds with Tyr745 and Gly582 of the HDAC6 protein, improving the tight binding. Additionally, Zn^2+^ of the HDAC6 protein was chelated by the ZBG of **STY12**. Moreover, the small molecule engaged in hydrophobic interactions with several residues, such as His463 and Phe583, providing strong van der Waals forces that stabilized the complex. The docking score of −7.324 kcal/mol suggested that the small molecule had a favorable binding affinity toward HDAC6.

**Figure 9:**
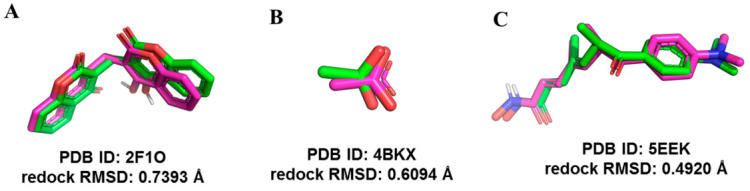
Superimposition of docking conformation and crystal conformation. (**A**) NQO1. (**B**) HDAC1. (**C**) HDAC6. Magenta for docking conformation, green for crystal conformation.

To validate the docking results, we performed a re-docking study using the crystal structure with HDAC1 (PDB: 4BKX), HDAC6 (PDB: 5EEI), and NQO1 (PDB ID: 2F1O), respectively, in which the co-crystallized ligand was re-docked into the protein binding pocket. The resulting docking conformation was then superimposed with the original crystal ligand conformation (Figure 9). The calculated RMSD was 0.6094 Å, 0.4920 Å and 0.7393 Å, respectively, indicating a high docking accuracy and suggesting that our docking method is reliable and the results are trustworthy.

### 3.7. **STY12** Exerted Its Anti-Cancer Effect Through ROS Production, Resulting in Cell Death

**Figure 10:**
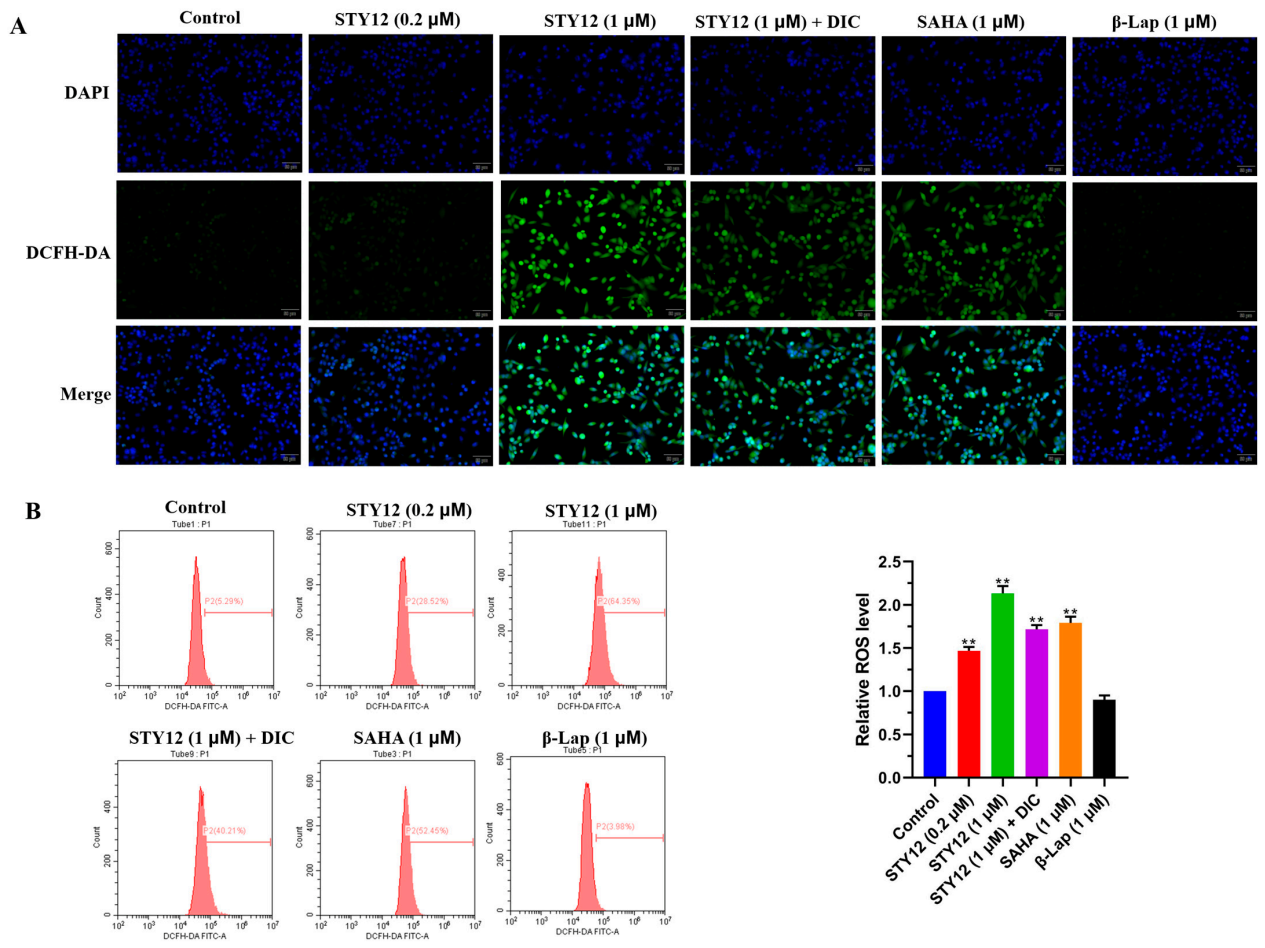
Compounds **STY12** (0.2, 1 μM), **STY12** (1 μM) + DIC (50 μM), SAHA (1 μM), and β-Lap (1 μM) increased the ROS levels in MIA PaCa-2 cells. (**A**) Fluorescence microscope, scale: 200/1. (**B**) Flow cytometry. Cells were treated for 48 h. The results are expressed as means ± SD. ** *p* < 0.01 vs. the control group.

**STY12** has high reduction rates via NQO1 and HDAC-inhibiting activity and is hypothesized to exert its antitumor effects via NQO1-mediated redox cycling and HDAC inhibition, thereby producing ROS while inducing cell death. Therefore, we first assessed ROS production in MIA PaCa-2 cells following exposure to **STY12**. The ROS contents were measured using a 2′,7′-dichlorfluorescein diacetate (DCFH-DA) redox-sensitive fluorescent probe and observed via fluorescence microscopy and flow cytometry. Exposure to **STY12** considerably increased the ROS content in MIA PaCa-2 cells in a dose-dependent manner (Figure 10). However, the **STY12**-induced increase in the ROS content was partially reduced by DIC (an NQO1 inhibitor), which suggested that this process was partially mediated by NQO1.

**Figure 11:**
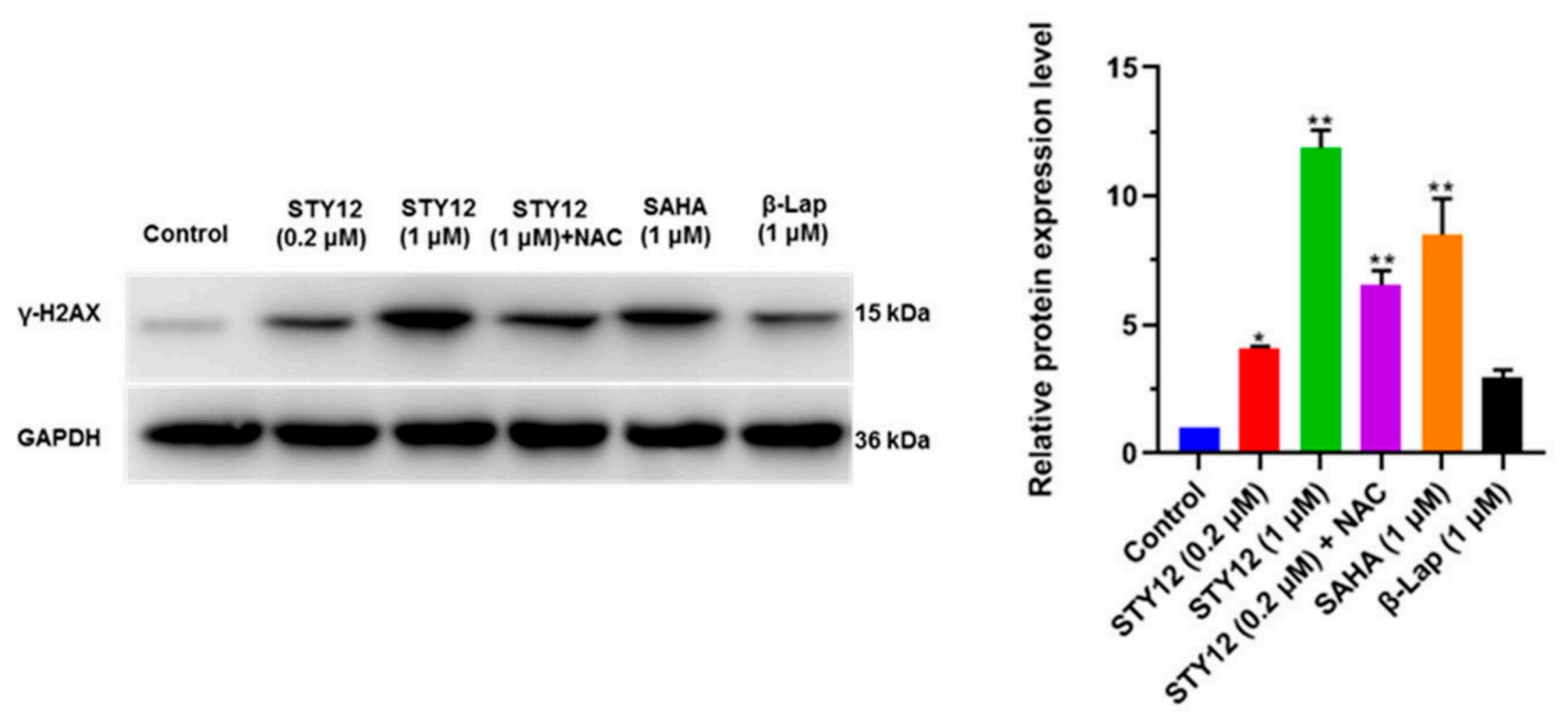
Compound **STY12** induced the upregulation of γ-H2AX in MIA PaCa-2 cells. Cells were treated with **STY12** (0.2, 1 μM), **STY12** (1 μM) + NAC (3 mM), SAHA (1 μM) and β-Lap (1 μM) for 48 h. GADPH: Glyceraldehyde-3-phosphatedehydrogenase; the results are expressed as the means ± SD. * *p* < 0.05, ** *p* < 0.01 vs. the control group. Original western blots can be found at Supplementary Materials.

**Figure 12:**
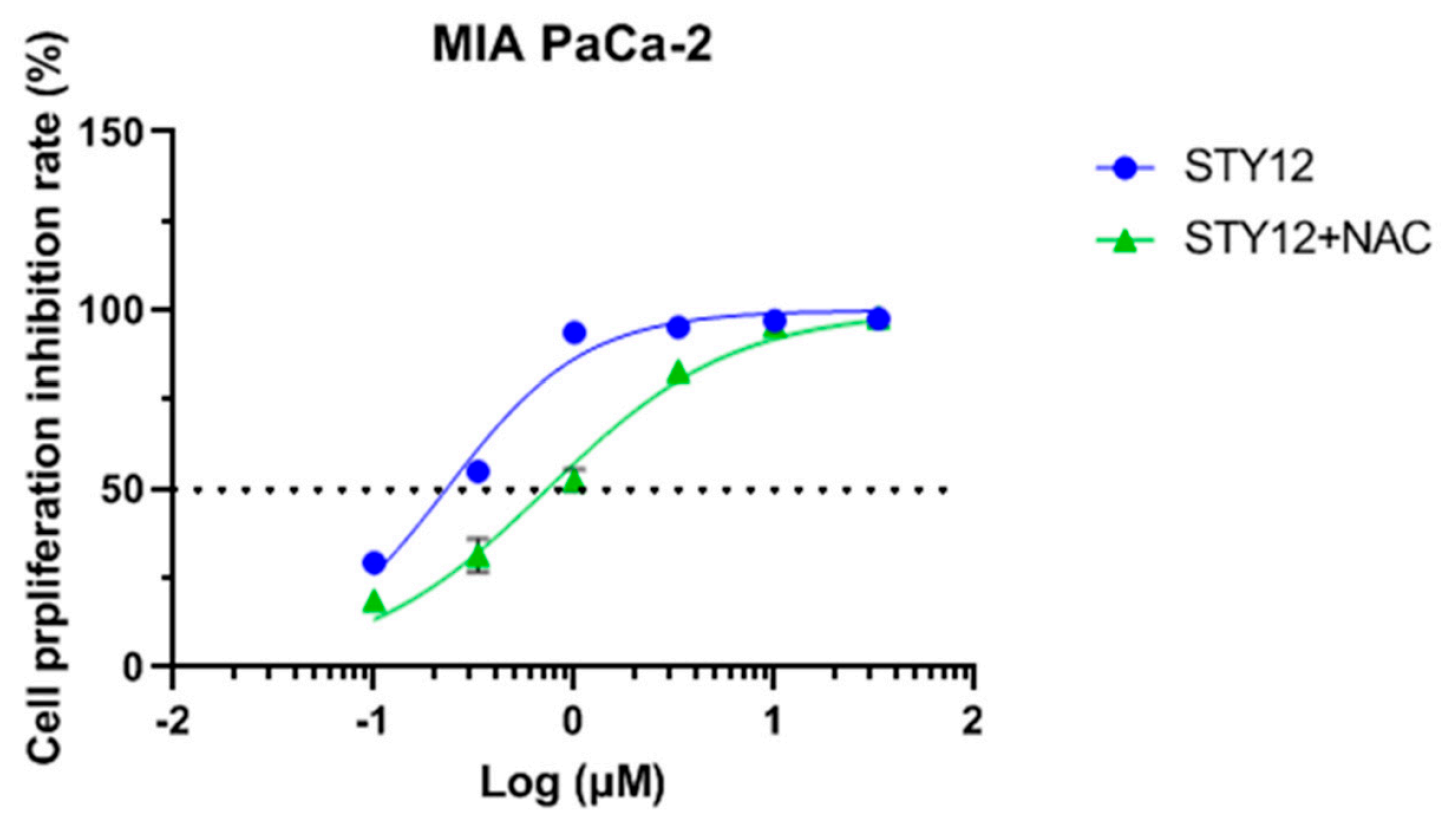
In vitro anti-proliferative activities of compounds **STY12** and **STY12**+NAC against MIA PaCa-2 cells. MIA PaCa-2 cells were treated with **STY12** (0.01–33 μM) and **STY12** (0.01–33 μM) + NAC (3 mM) for 72 h. Data were analyzed using GraphPad Prism 8.0, and results are expressed as the means ± SD of at least two separate determinations.

The accumulation of high levels of ROS irreversibly damages DNA and ultimately leads to cell death. Therefore, we measured DNA damage by evaluating histone H2AX phosphorylation (γ-H2AX, a DNA damage marker) in **STY12**-treated MIA PaCa-2 cells. **STY12** treatment induced severe DNA damage, which was substantially alleviated in *N*-acetylcysteine (NAC)-pretreated MIA PaCa-2 cells (Figure 11). Additionally, after the MIA PaCa-2 cells were pretreated with 3 mM NAC before they were treated with **STY12**, **STY12** also exerted weaker cytotoxic effects (IC_50_ = 0.73 ± 0.02) (Figure 12). These results indicate that **STY12** exerts its antitumor effects via NQO1-mediated redox cycling and HDAC inhibition, leading to ROS generation and cell death.

### 3.8. **STY12** Induced Cell Apoptosis in MIA PaCa-2 Cells

**Figure 13:**
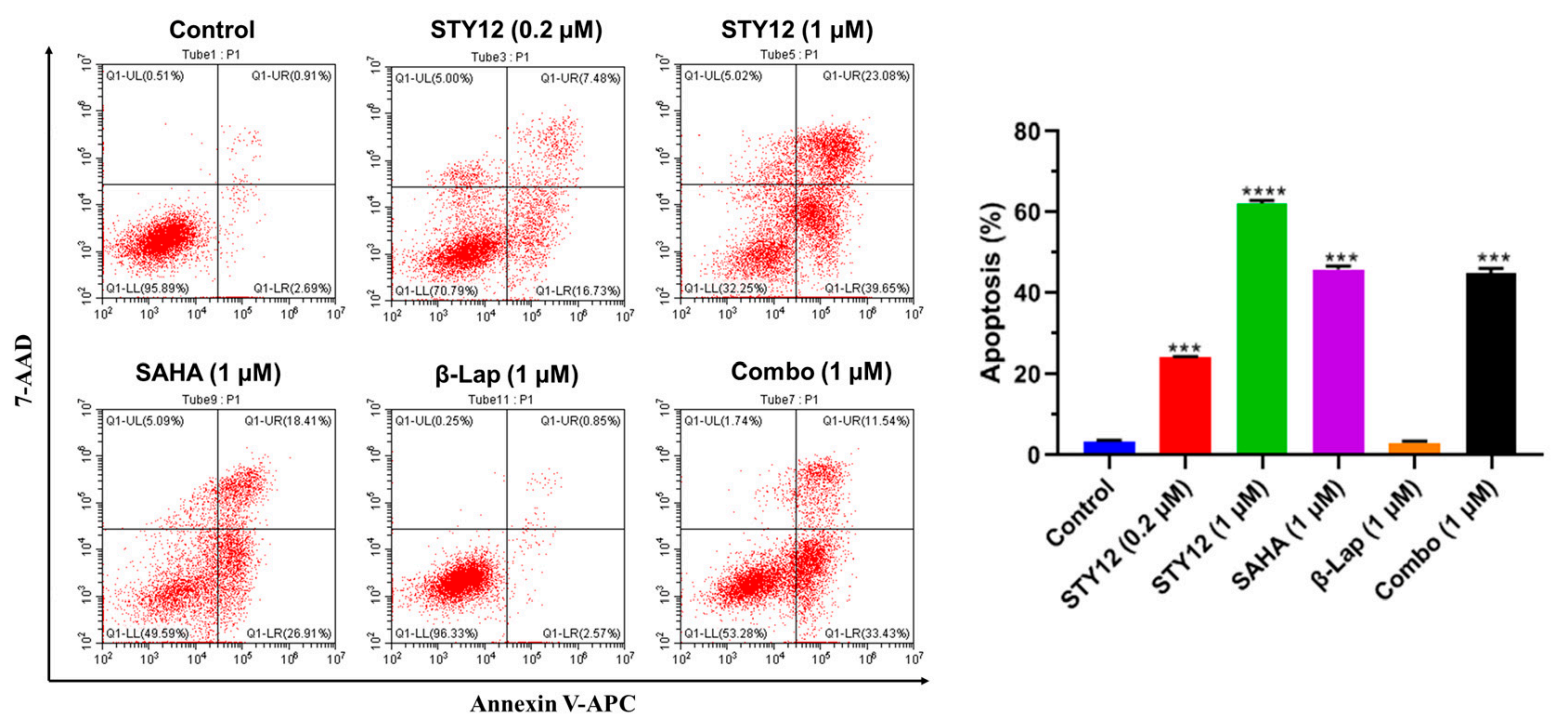
Compound **STY12** induced cell apoptosis in MIA PaCa-2 cells. Cells were treated with **STY12** (0.2, 1 μM), SAHA (1 μM), β-Lap and SAHA (1 μM) + β-Lap (1 μM) (Combo) for 48 h. The results are expressed as means ± SD. *** *p* < 0.001 and **** *p* < 0.0001 vs. control.

Constitutive DNA damage induces apoptosis; therefore, Annexin V-FITC/7-AAD double-staining was conducted to analyze how **STY12** affects apoptosis. MIA PaCa-2 cells were subjected to 48 h of **STY12** treatment before flow cytometry assays were conducted. **STY12** strongly promoted PC apoptosis in a dose-dependent manner (Figure 13). Treatment with 1 μM **STY12** at a concentration of 62.73% dramatically accelerated the apoptosis of MIA PaCa-2 cells.

### 3.9. **STY12** Induced Cell Cycle Arrest in MIA PaCa-2 Cells

**Figure 14:**
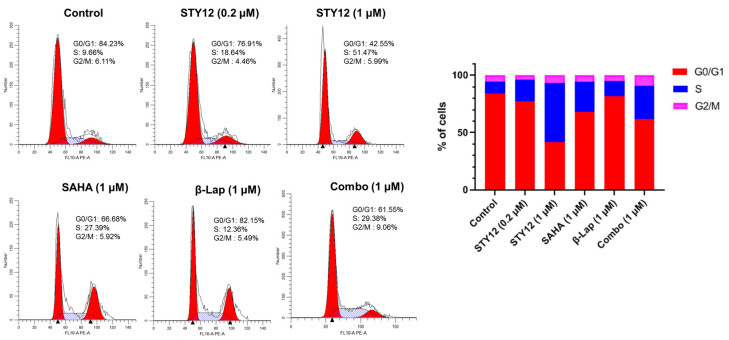
Compound **STY12** induced cell cycle arrest in MIA PaCa-2 cells. Cells were treated with **STY12** (0.2, 1 μM), SAHA (1 μM), β-Lap and SAHA (1 μM) + β-Lap (1 μM) (Combo) for 48 h. The results are expressed as the means.

Disturbances in the cell cycle are common during the occurrence of several tumors. Flow cytometry assays were performed to evaluate how **STY12** affects the progression of the cell cycle in MIA PaCa-2 cells (Figure 14). After MIA PaCa-2 cells were treated for 48 h with 0, 0.2, and 5 μM **STY12**, the proportion of cells in the S phase increased from 9.66% to 51.47%, whereas the proportion of cells in the G0/G1-phase decreased. Therefore, compared with SAHA and β-Lap, **STY12** strongly arrested MIA PaCa-2 cells in the S phase in a dose-dependent manner and was more effective at inducing cell cycle arrest at the S phase.

### 3.10. **STY12** Inhibited the Invasion of MIA PaCa-2 Cells

**Figure 15:**
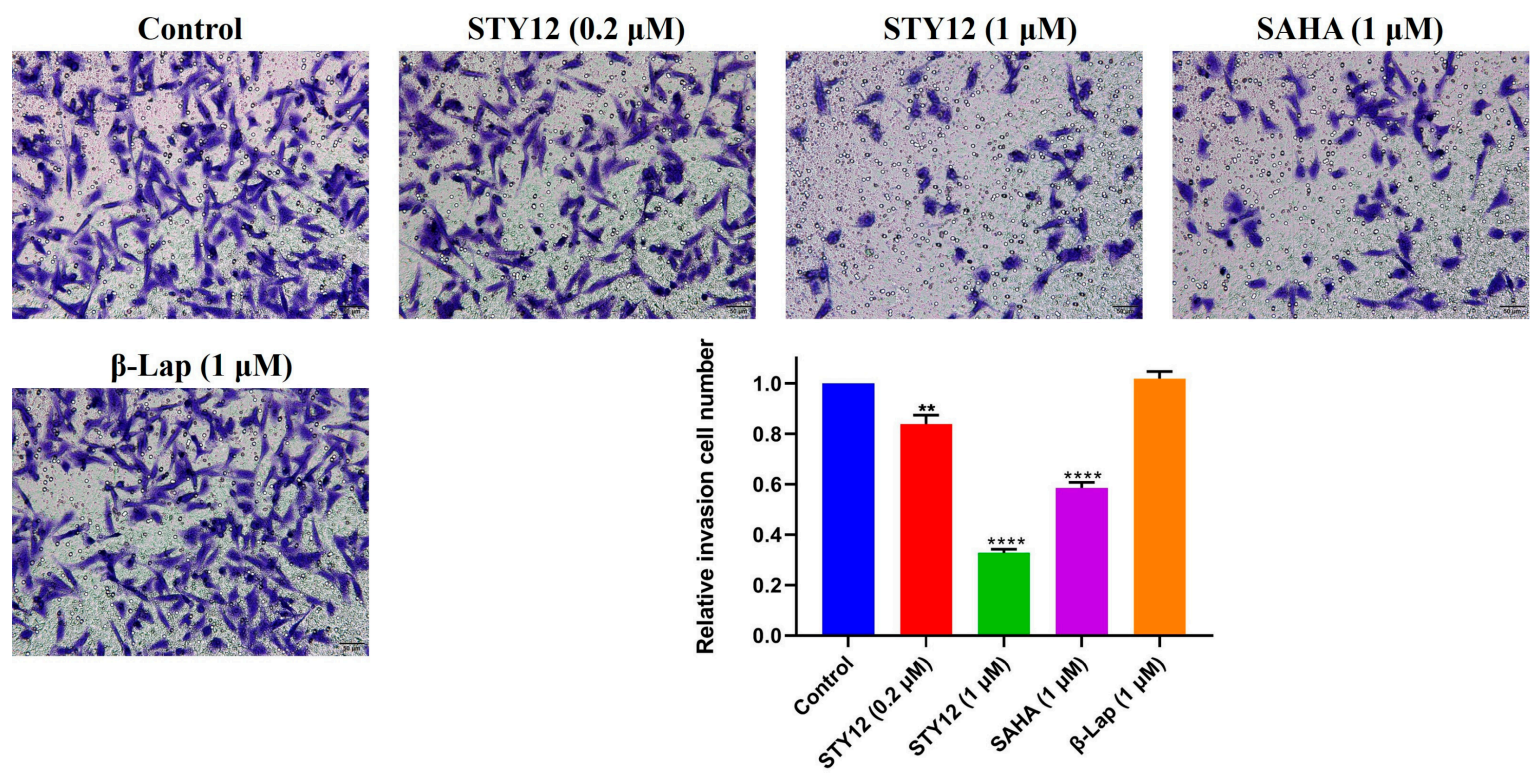
**STY12** suppressed the invasion of MIA PaCa-2 cells. Cells were treated with **STY12** (0.2, 1 μM), SAHA (1 μM) and β-Lap (1 μM) for 48 h. Scale: 200/1. The results are expressed as means ± SD. ** *p* < 0.01 and **** *p* < 0.001 vs. control.

PC has a potent metastatic capacity, resulting in an unfavorable prognostic outcome. Therefore, the ability of **STY12** to resist the metastasis of MIA PaCa-2 cells was analyzed by performing a Transwell assay. After MIA PaCa-2 cells were treated with **STY12** at the indicated concentrations, invasion was blocked in a dose-dependent manner (Figure 15). Moreover, compared with SAHA and β-Lap, **STY12** also showed greater antimetastatic activity.

### 3.11. In Vivo Antitumor Efficacy of **STY12**

**Figure 16:**
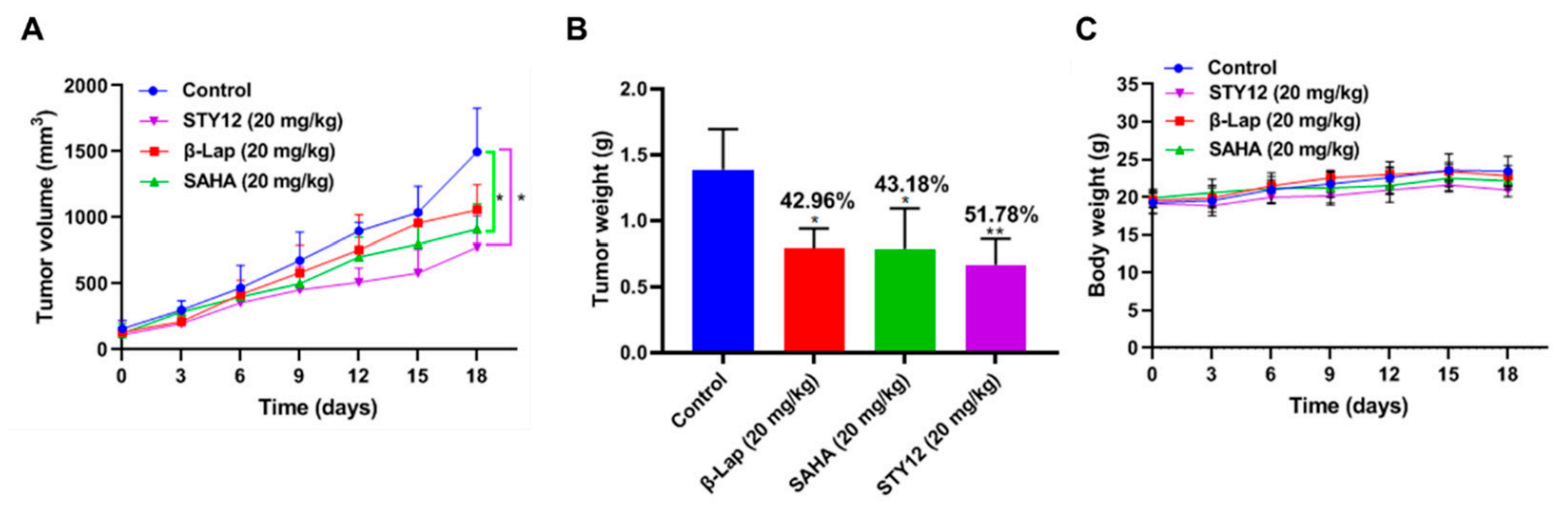
In vivo antitumor efficacy of compound **STY12** in the MIA PaCa-2 tumor xenograft model. (**A**) Tumor volume changes during treatment with **STY12**. (**B**) Tumor weight after treatment with **STY12**. (**C**) Body weight changes during treatment with **STY12**. All the data are expressed as the means ± SD (n = 4). * *p* < 0.05, ** *p* < 0.01 vs. the control group.

To determine the in vivo antitumor effects of **STY12**, a MIA PaCa-2 xenograft nude mouse model was constructed. When the mean tumor volume reached about 150 mm^3^, the animals were randomly classified into four groups and intraperitoneally administered control solvent, 20 mg/kg β-Lap, or 20 mg/kg **STY12** every other day for 18 days. After 18 days, treatment with 20 mg/kg **STY12** significantly reduced the growth of the MIA PaCa-2 tumors (TGI = 51.78%), outperforming the positive controls (Figure 16). Moreover, body weight did not decrease significantly after exposure to **STY12**. Overall, **STY12** had strong in vivo antitumor effects on MIA PaCa-2 cells without any prominent toxic effects.

## 4. Discussion

**Figure 17:**
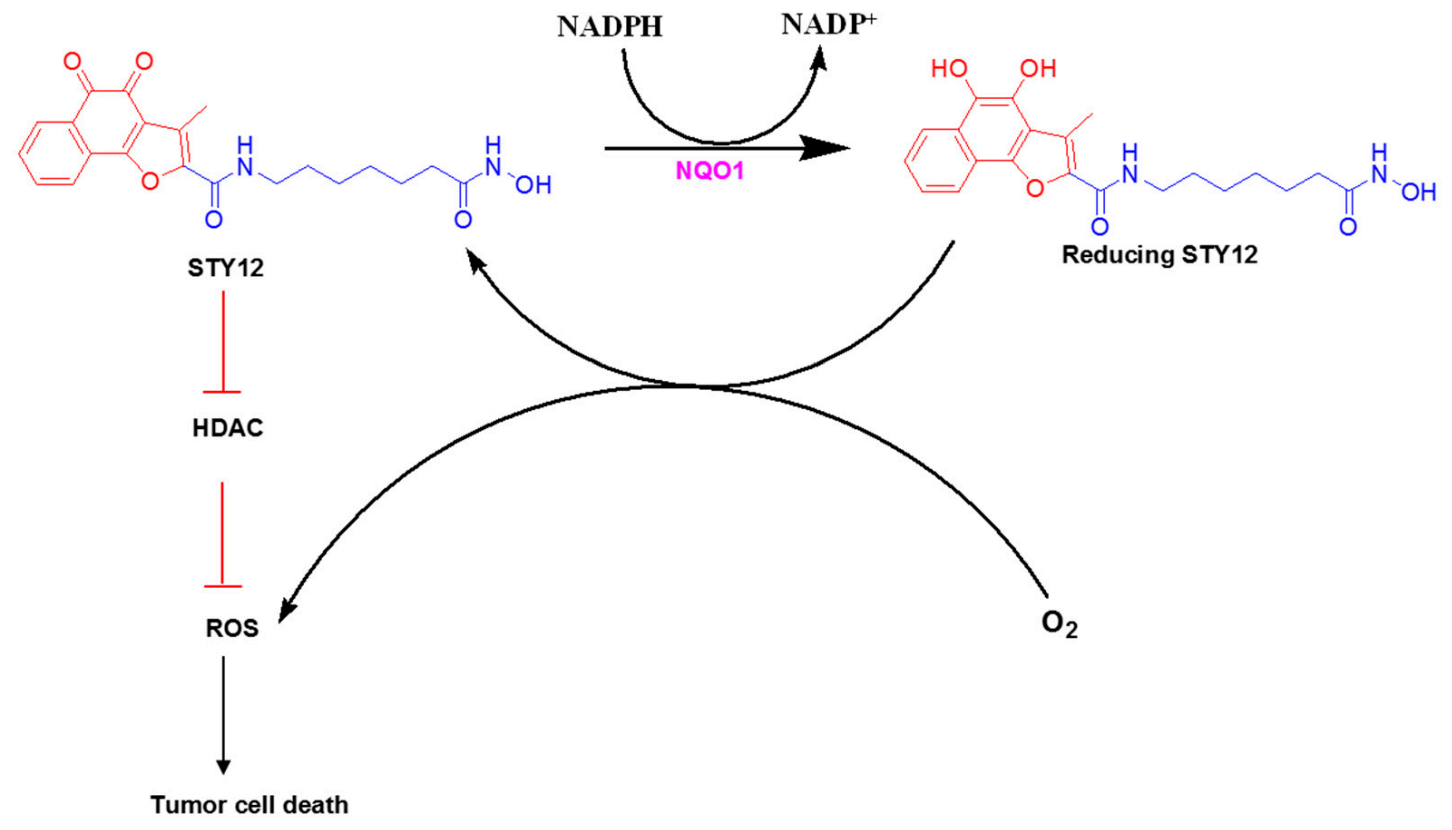
Proposed mechanism of action of **STY12**.

Cancer is a complex disease, and a single treatment cannot meet the clinical needs. In addition, long-term excessive activation or inhibition of a specific target can lead to drug resistance and even toxic adverse effects. In recent years, multi-target drugs have garnered significant attention in the development of anti-cancer therapeutics. Compared with drug combinations and single-target drugs, multi-target drugs may offer advantages such as improved patient compliance, reduced drug–drug interactions and drug resistance, and simplified pharmacokinetics [43], based on the synergistic effect of β-Lap and SAHA on PC. In the study, we developed a novel NQO1/HDAC dual-targeting agent using a pharmacophore fusion strategy. **STY12** exhibited excellent reduction rates by NQO1 and can rapidly generate large amounts of toxic ROS via redox cycling of hydroquinones with the corresponding quinone substrates in NQO1-overexpressing cells. Moreover, the compound strongly inhibited HDAC1 and HDAC6. Mechanistic studies revealed that **STY12** promoted H3 and α-tubulin acetylation and ROS production to exert antitumor effects (Figure 17). Moreover, **STY12** showed good anti-proliferative effects against PC MIA PaCa-2, SW1990, and Capan-2 cells. However, similar to many quinone drugs, compound **STY12** also showed submicromolar activity against normal cell lines, indicating a lower safety profile. Thus, enhancing its safety through structural modifications is essential.

**Figure 18:**
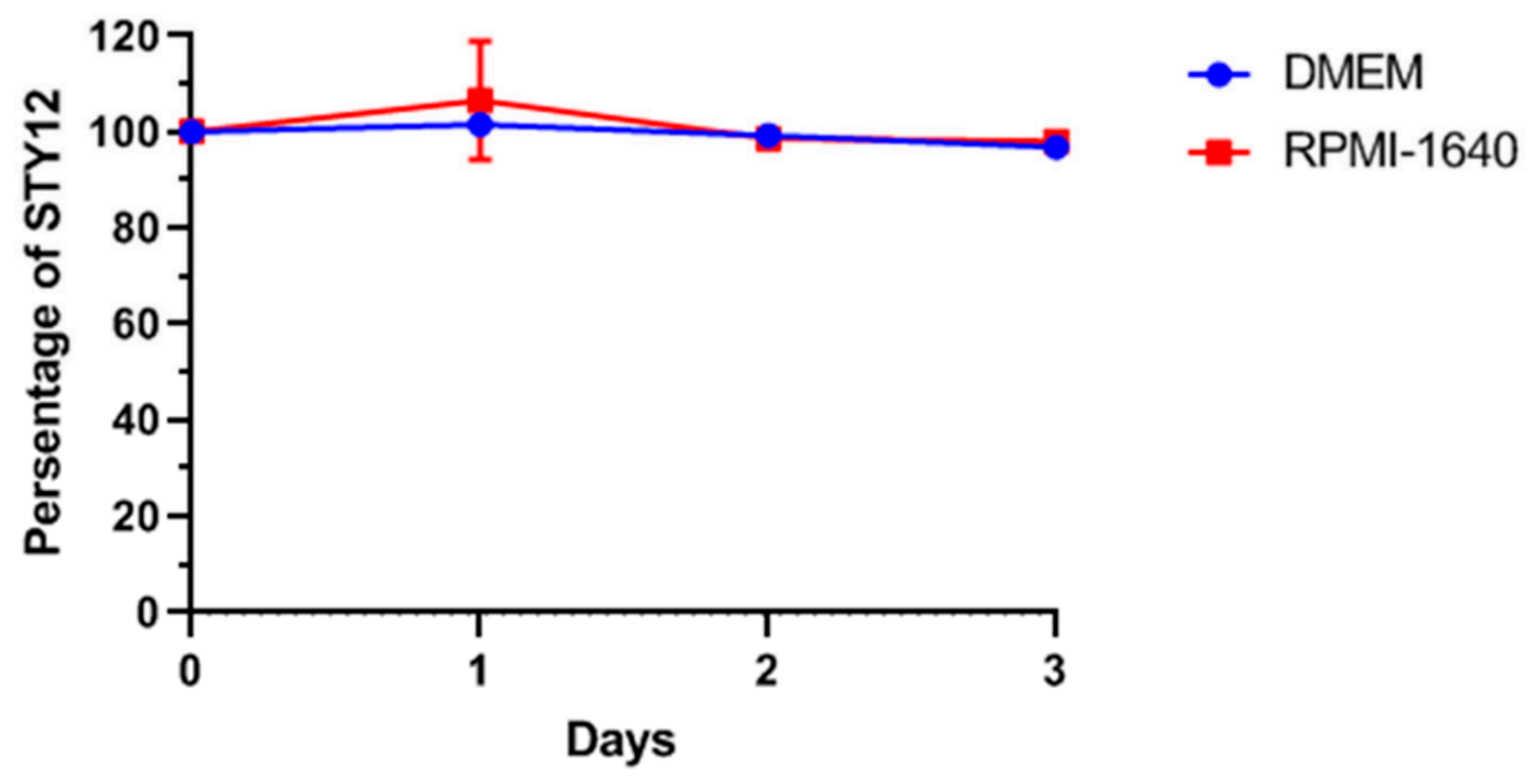
Stability of **STY12** in cell culture media.

Through a comprehensive in silico physicochemical analysis (Table 3), it was found that **STY12** exhibits good gastrointestinal (GI) absorption and desirable drug-likeness criteria. However, the compound also showed poor aqueous solubility and blood–brain barrier permeability (BBB). Moreover, compound **STY12** also possessed good stability in cell culture media. After incubation with cell culture media for 3 days, the content of **STY12** did not significantly decrease (Figure 18).

Despite the promising efficacy, two challenges continue to hinder the translation of **STY12** into clinical practice. One of the most significant obstacles is the issue of safety and toxicity. **STY12** is not only an effective NQO1 substrate, but can also be triggered by cytochrome p450 reductase (CPR), and induce hepatotoxicity. Pharmacokinetic complexity presents yet another hurdle. **STY12** showed poor aqueous solubility and BBB. Future studies will focus on structural optimization of **STY12** to improve safety and solubility.

## 5. Conclusions

In this study, we report a novel NQO1/HDAC dual-targeting agent, **STY12**, which exhibited potent anti-pancreatic cancer activity through ROS-mediated DNA damage. **STY12** strongly affected the PC cell lines MIA PaCa-2, SW1990, and Capan-2 in vitro, with IC_50_ values of 0.23 ± 0.01, 0.25 ± 0.01 and 0.14 ± 0.02 μM, respectively. **STY12** also strongly inhibited HDAC1 and HDAC6 activity (IC_50_ = 29 and 10 nM, respectively) and exhibited excellent reduction rates by NQO1 (kcat/Km = 5.59 × 10^6^ M^−1^s^−1^). Mechanistic studies revealed that **STY12** promoted H3 and α-tubulin acetylation and ROS production to exert antitumor effects. Additionally, **STY12** arrested the cell cycle at the S phase, suppressed invasion, and increased apoptosis in MIA PaCa-2 cells. Additionally, compared with SAHA and β-Lap, **STY12** exhibited strong in vivo antitumor effects, with negligible toxic effects. Considering the advantages of **STY12**, the dual-targeting agent may be used as an anti-PC agent.

## Figures and Tables

**Table 1:** In vitro anti-proliferative activities of compound **STY12**.

Compd.	IC_50_ (μM) ^a^				
	MIA PaCa-2	SW1990	Capan-2	hTERT-HPNE	BEAS-2B
**STY12**	0.23 ± 0.01	0.25 ± 0.01	0.14 ± 0.02	0.83 ± 0.08	1.78 ± 0.26
SAHA	1.03 ± 0.07	8.47 ± 4.24	1.53 ± 0.03	3.62 ± 0.48	1.09 ± 0.08
β-Lap	2.48 ± 0.56	0.29 ± 0.09	1.26 ± 0.06	1.35 ± 0.06	4.74 ± 0.44
Combo	0.77 ± 0.16	0.96 ± 0.19	1.21 ± 0.06	1.17 ± 0.06	1.30 ± 0.02

^a^ The IC_50_ values were calculated after cell incubation with the tested compounds (0.01–33 μM) for 72 h. Data were analyzed using GraphPad Prism 8.0, and results are expressed as the means ± SD of at least two separate determinations.

**Table 2:** Inhibitory effect of **STY12** toward different HDAC isoforms ^a^.

HDAC Class	HDAC Isoform	STY12 (IC_50_, nM)	SAHA (IC_50_, nM)
I	HDAC1	29 ± 8	35 ± 4
	HDAC8	1672 ± 578	3303 ± 359
IIA	HDAC4	19,461 ± 4882	66,646 ± 1269
IIB	HDAC6	10 ± 3	11 ± 1
IV	HDAC11	5383 ± 1667	34,386 ± 5162

^a^ The IC_50_ values were determined with nonlinear regression through a normalized dose–response fit using the GraphPad Prism 8, and results are expressed as the means ± SD of at least two separate determinations.

**Table 3:** ADME pharmacokinetics and drug-likeness parameters of **STY12**.

Physicochemical Properties	Pharmacokinetics	Druglikeness
Formula: C_21_H_21_NO_7_	GI absorption: High	Lipinski Yes; 0 violation
Molecular weight: 399.39 g/mol	BBB permeant: No	Ghose: Yes
Num. heavy atoms: 29	P-gp substrate: Yes	Veber: Yes
Num. arom. heavy atoms: 11	CYP1A2 inhibitor: No	Egan: Yes
Fraction Csp3: 0.33	CYP2C19 inhibitor: No	Muegge: Yes
Num. rotatable bonds: 10	CYP2C9 inhibitor: Yes	Bioavailability Score: 0.55
Num. H-bond acceptors: 7	CYP2D6 inhibitor: No	
Num. H-bond donors: 2	CYP3A4 inhibitor: No	
Molar Refractivity: 101.62	Log Kp: −6.86 cm/s	
TPSA: 122.91 Å^2^		
Solubility: 1.96 × 10^−4^ mg/mL; 4.90 × 10^−7^ mol/L		

## Data Availability

The original contributions presented in this study are included in the article/Supplementary Materials. Further inquiries can be directed to the corresponding author.

## Supplementary Materials

The following supporting information can be downloaded at: https://www.mdpi.com/article/10.3390/biom16060812/s1. Figure S1. Full-length unprocessed blots for Figure 3. Figure S2. Full-length unprocessed blots for Figure 6. Figure S3. Full-length unprocessed blots for Figure 11. Figure S4. ^1^H NMR of **2**. Figure S5. HRMS of **2**. Figure S6. ^1^H NMR of **3**. Figure S7. HRMS of **3**. Figure S8. ^1^H NMR of **6**. Figure S9. HRMS of **6**. Figure S10. ^1^H NMR of **7**. Figure S11. HRMS of **7**. Figure S12. ^1^H NMR of **8**. Figure S13. ^13^CNMR of **8**. Figure S14. HRMS of **8**. Figure S15. ^1^H NMR of **STY12**. Figure S16. ^13^CNMR of **STY12**. Figure S17. HRMS of **STY12**. Figure S18. HPLC of **STY12**.
