# Supplementary material for: STY12, a Novel NQO1/HDAC Dual-Targeting Agent, Exhibits Potent Anti-Pancreatic Cancer Activity by ROS-Mediated DNA Damage
Source: Biomolecules. 2026 May 30;16(6):812. doi: 10.3390/biom16060812 (PMC13296467; doi:10.3390/biom16060812)
Supplement: Supplementary file 1 [file biomolecules-16-00812-s001.zip › biomolecules-4251606-supplementary.pdf]

Supplemental Figures

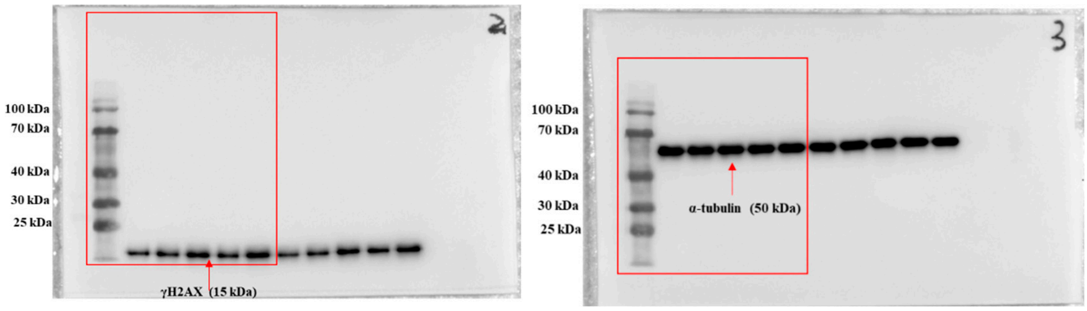

Figure S1. Full-length unprocessed blots for Figure 3.

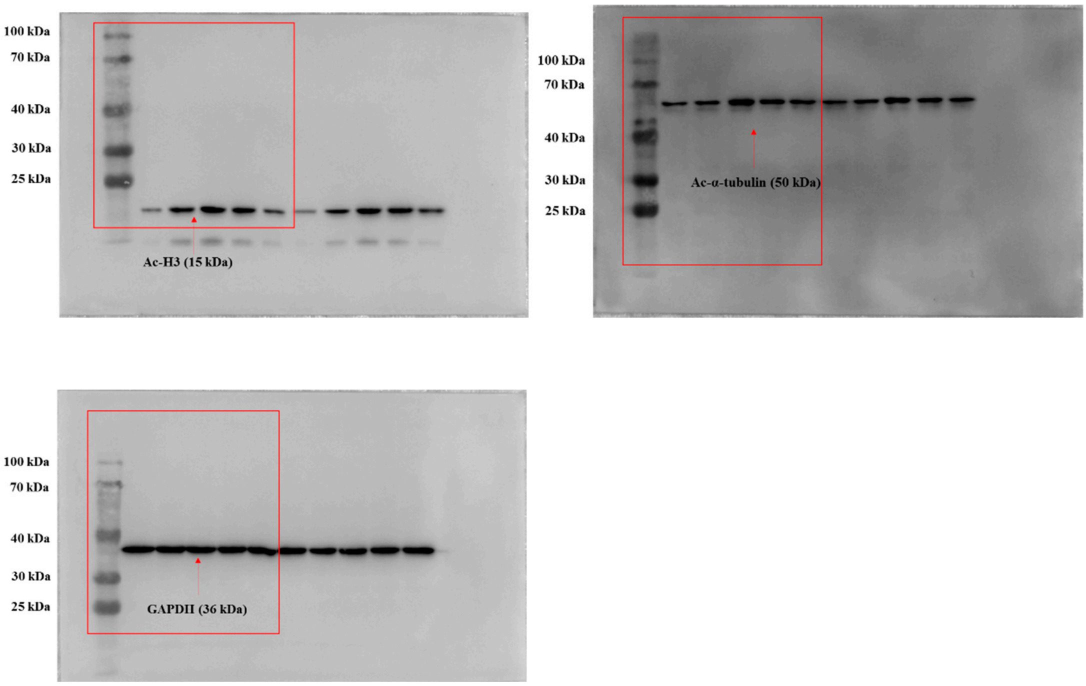

Figure S2. Full-length unprocessed blots for Figure 6.

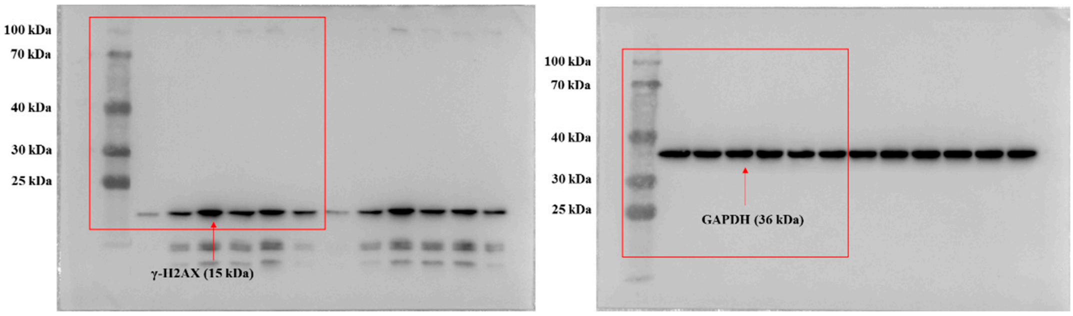

Figure S3. Full-length unprocessed blots for Figure 11.

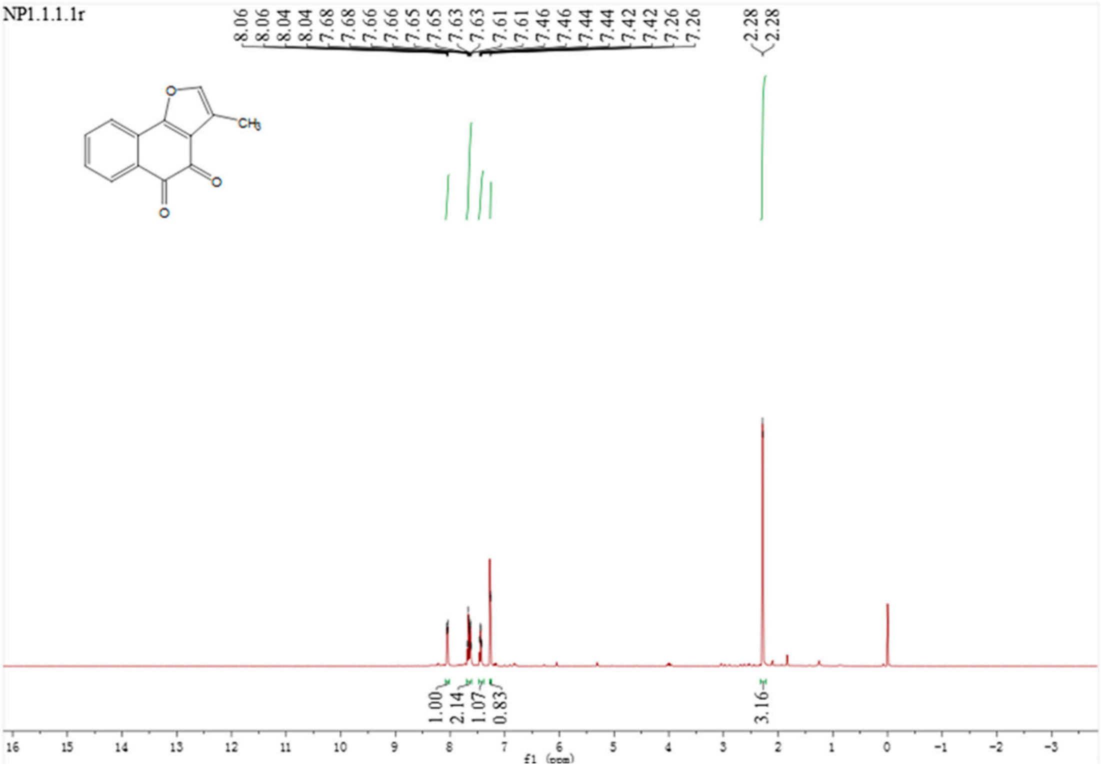

Figure S4. **[1]**H NMR of **2.**

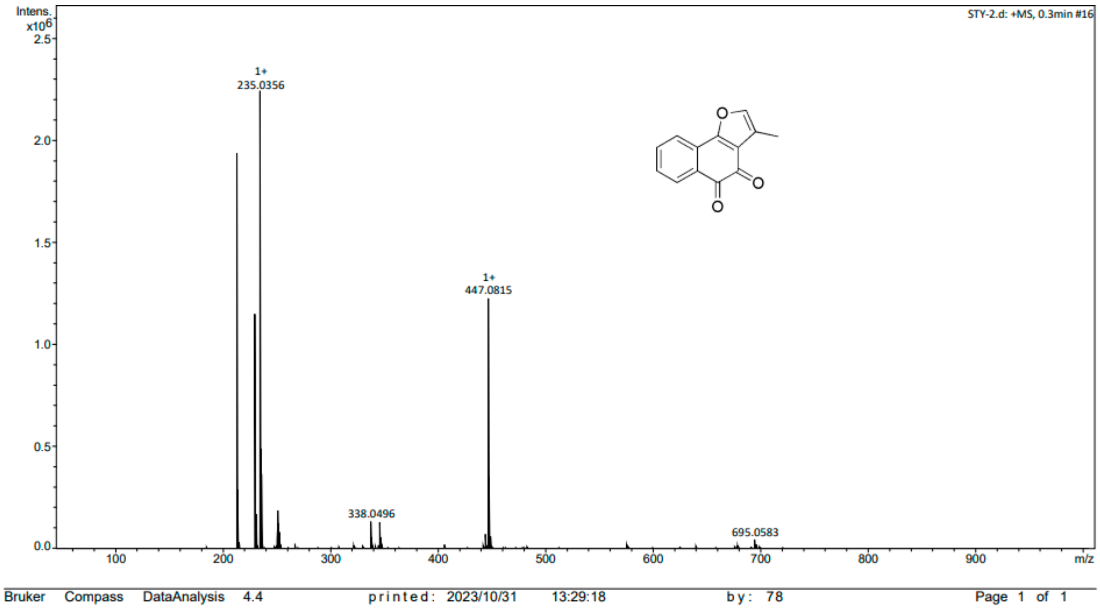

Figure S5. HRMS of **2.**

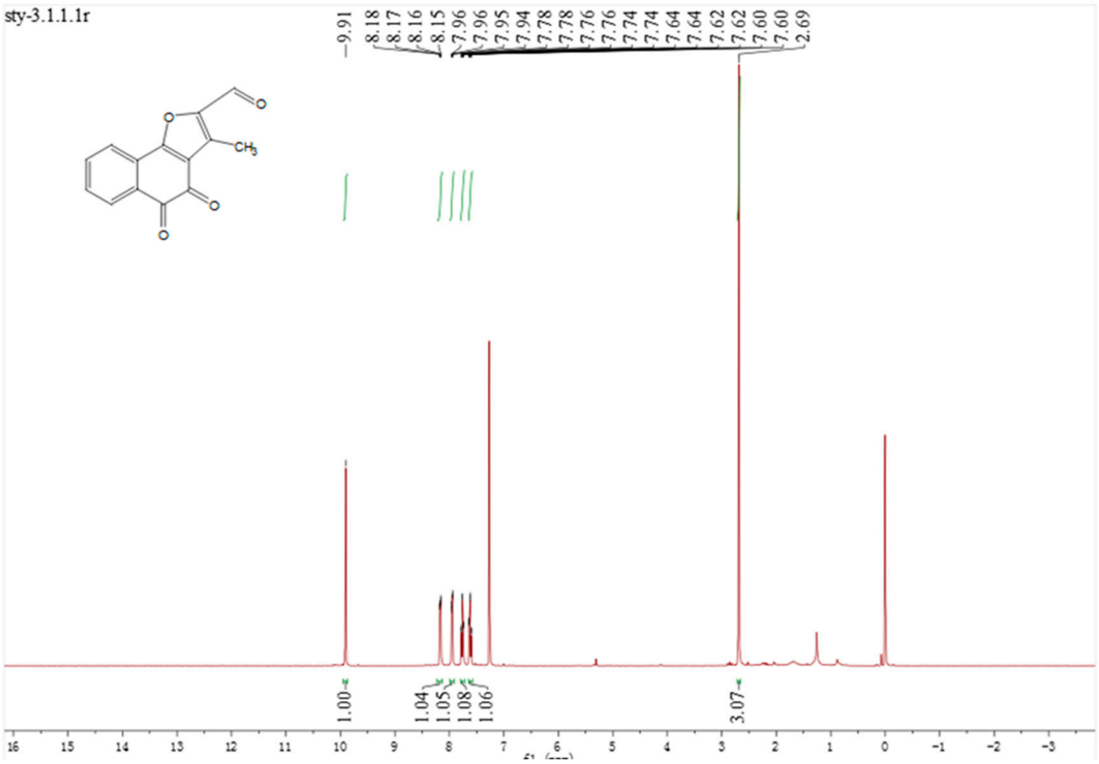

Figure S6. ^{1}H NMR of **3.**

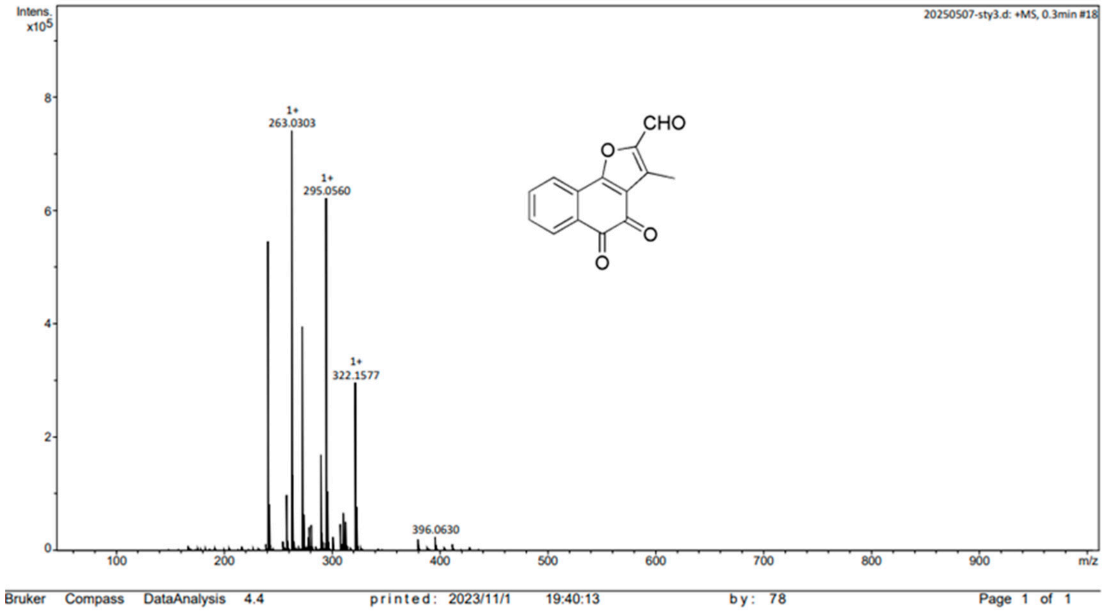

Figure S7. HRMS of **3.**

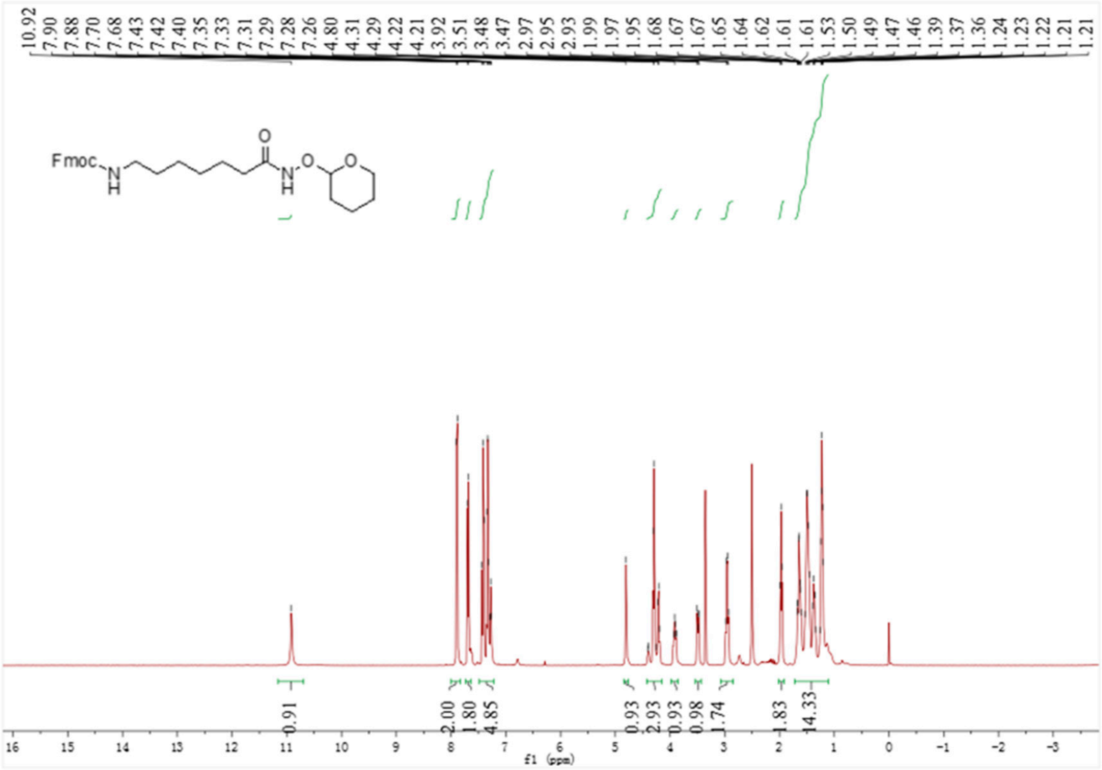

Figure S8. ^{1}H NMR of **6.**

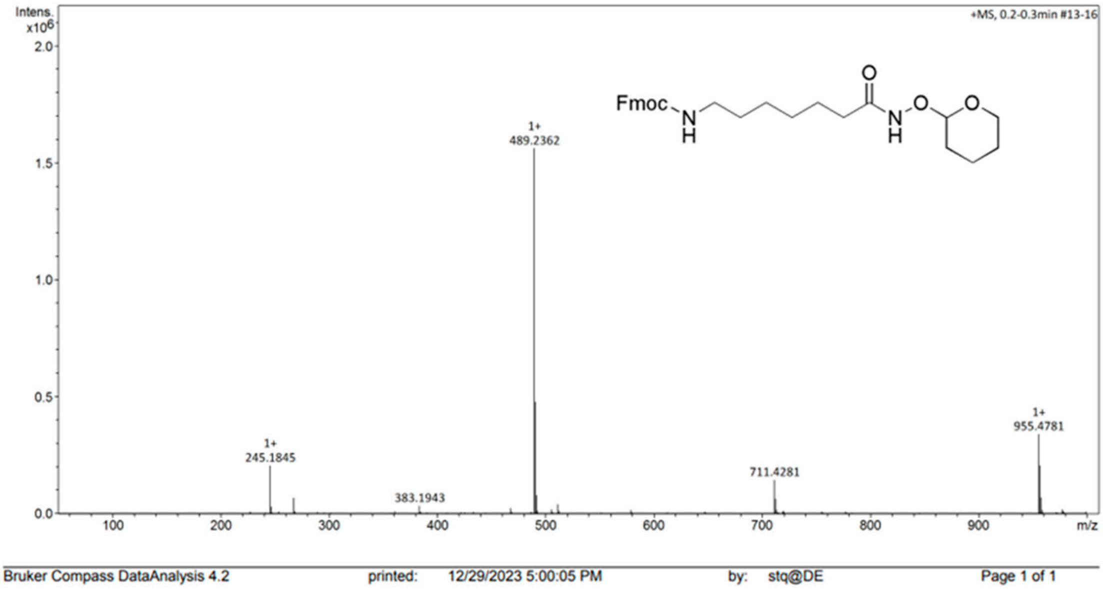

Figure S9. HRMS of **6.**

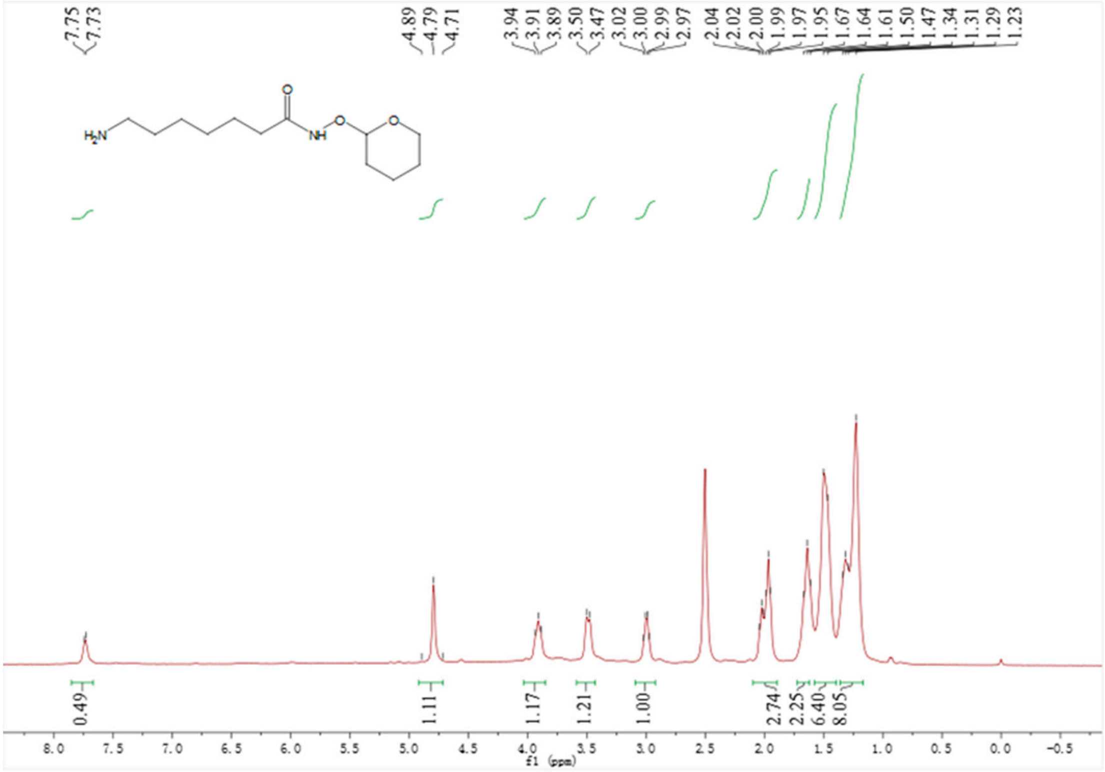

Figure S10. ^{1}H NMR of **7.**

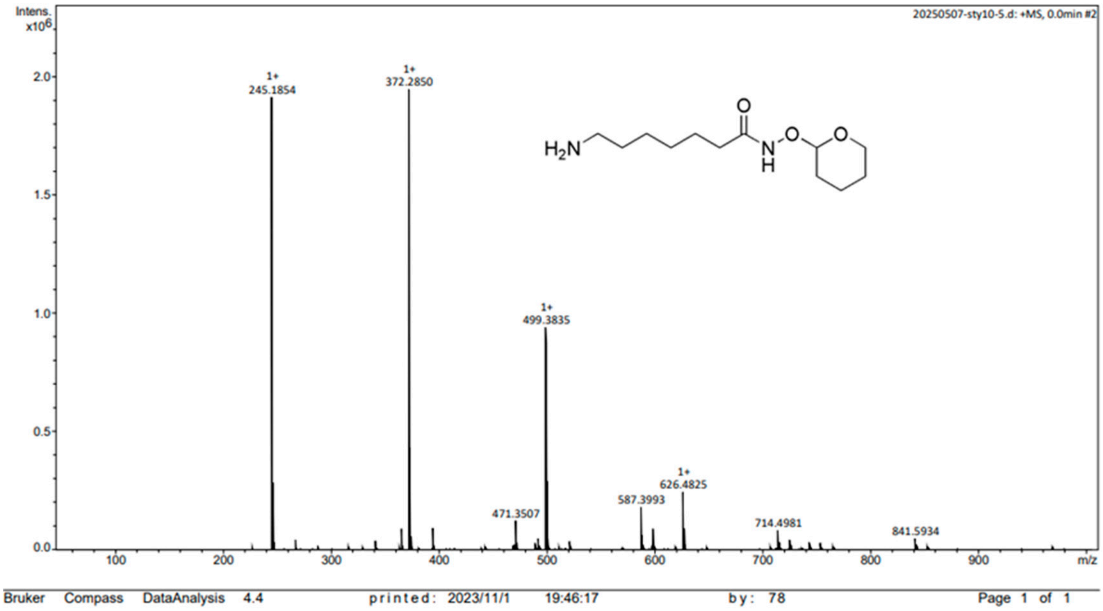

Figure S11. HRMS of **7.**

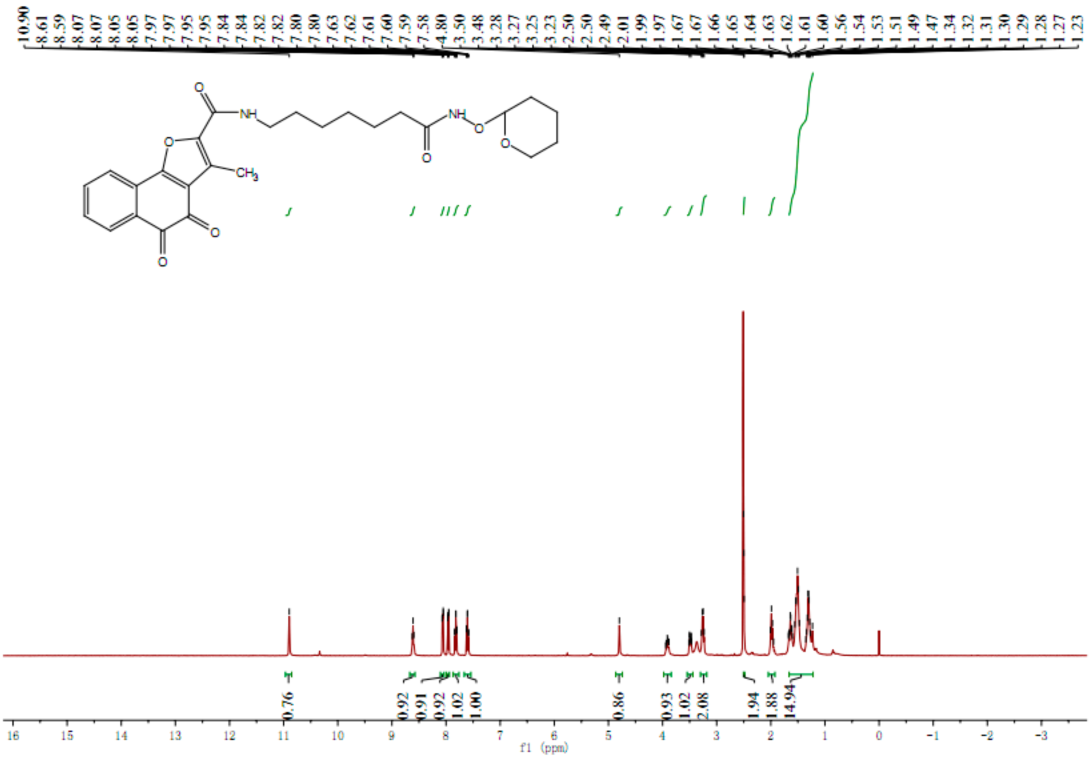

Figure S12. ^{1}HNMR of **8.**

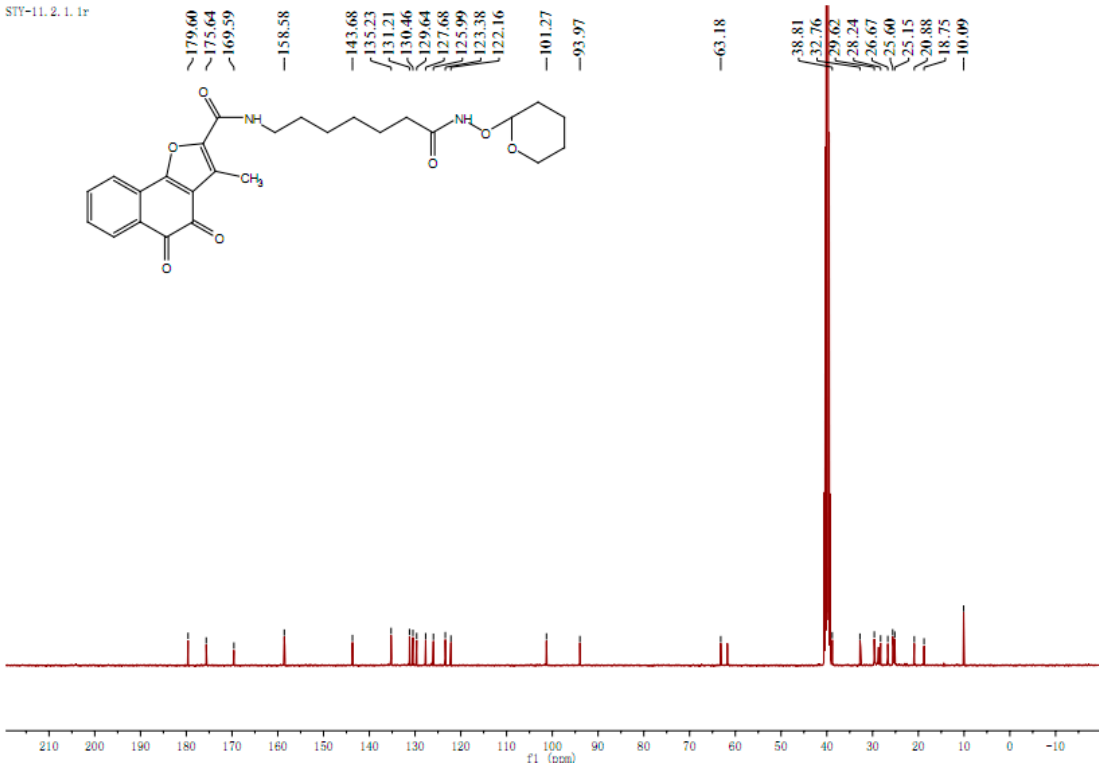

Figure S13. ^{13}CNMR of **8.**

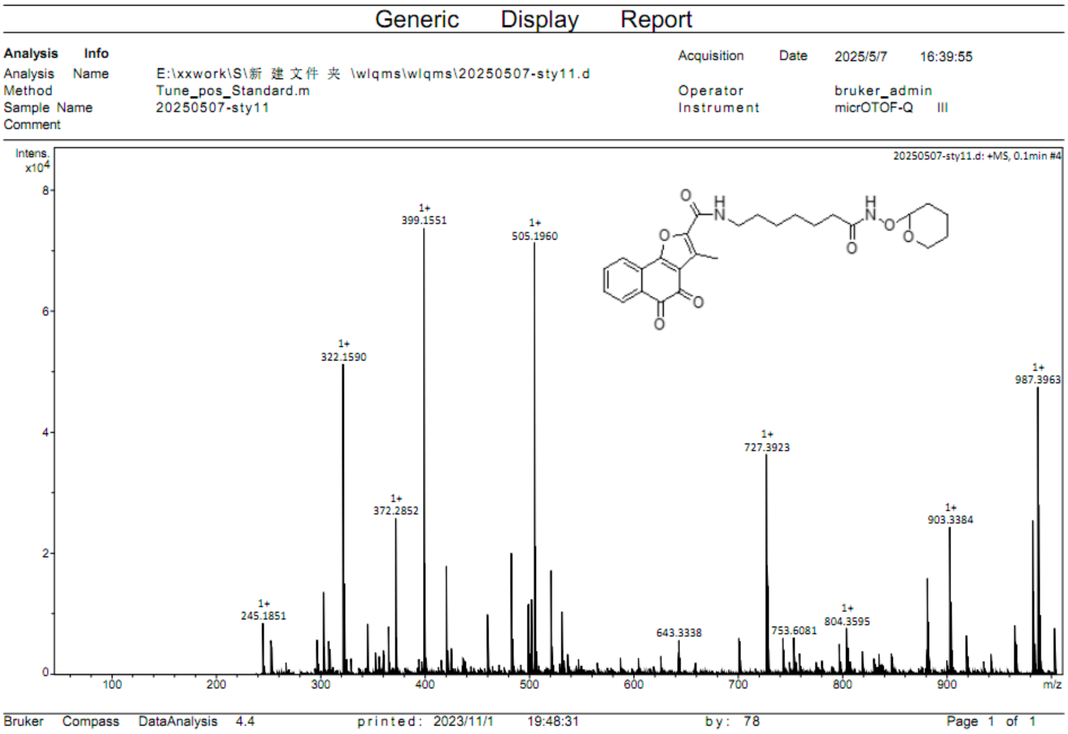

Figure S14. HRMS of **8.**

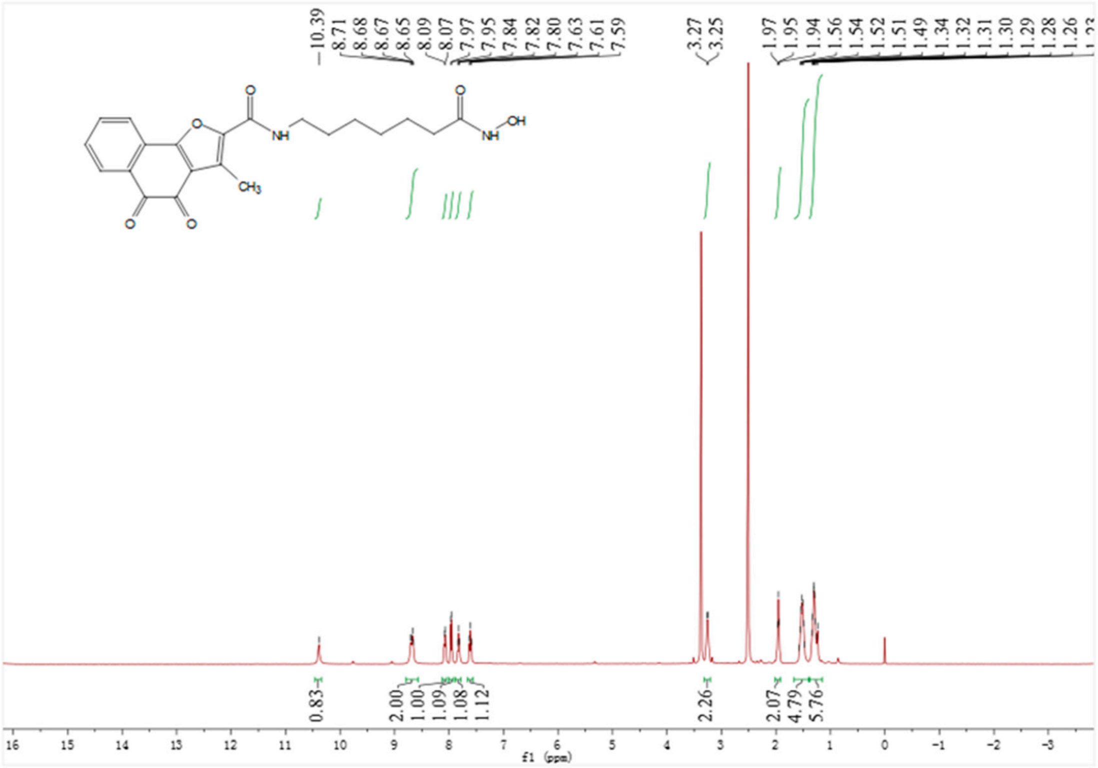

Figure S15. ^{1}H NMR of **STY12.**

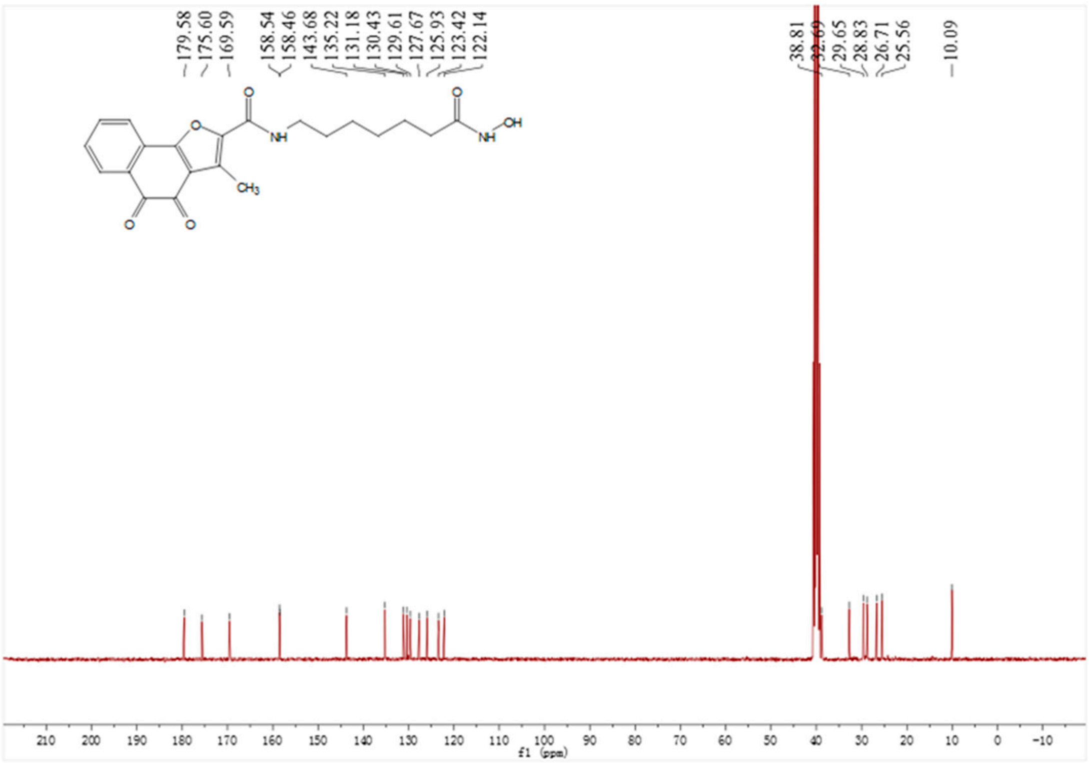

Figure S16. ^{13}C NMR of **STY12.**

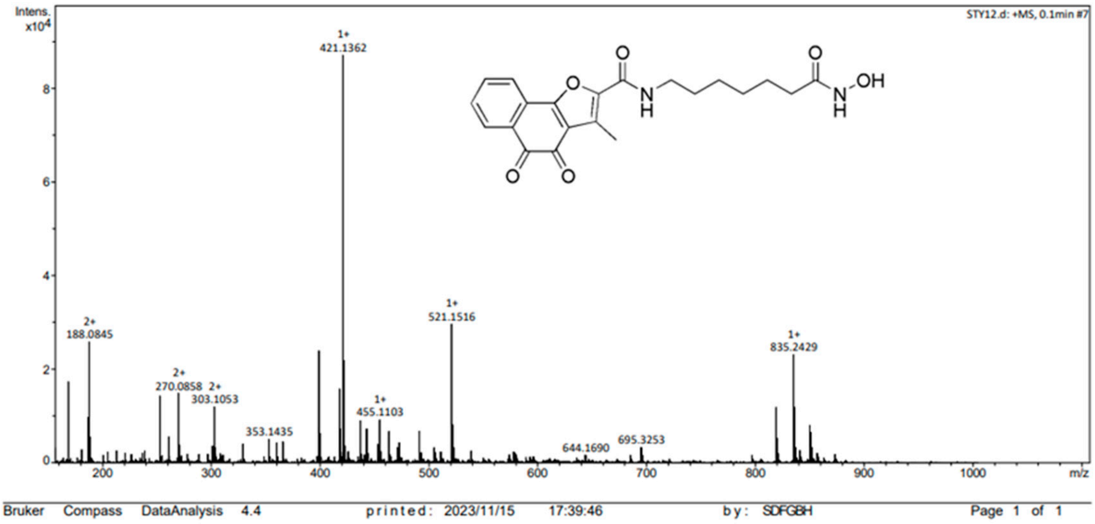

Figure S17. HRMS of **STY12.**

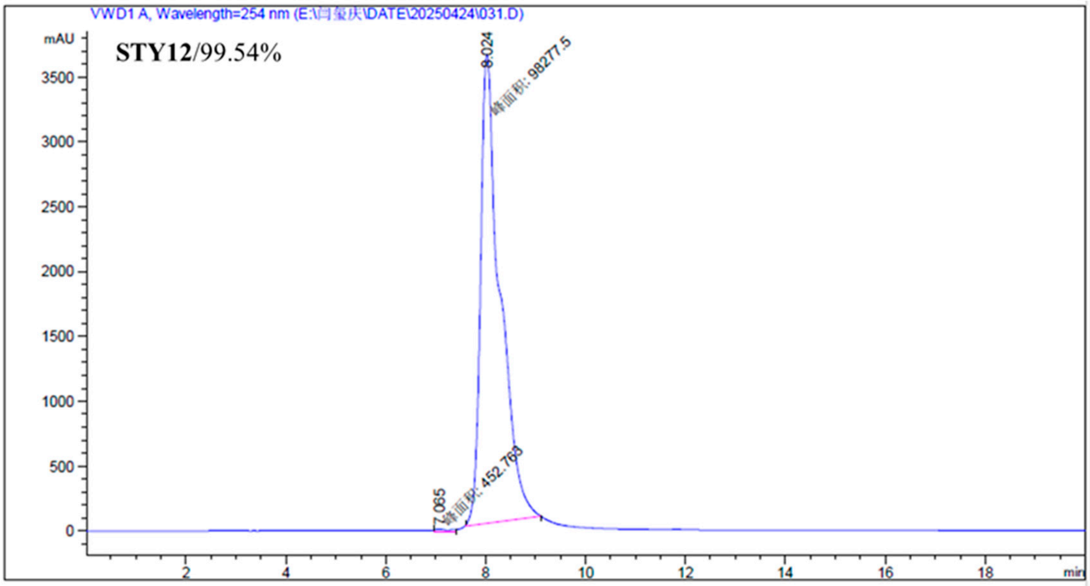

Figure S18. HPLC of **STY12.**
